# Can cognitive function tests discriminate between patients with glioma and healthy controls prior to treatment? A systematic review

**DOI:** 10.1371/journal.pone.0329663

**Published:** 2025-08-06

**Authors:** Laura Standen, Aiman Aslam, Roisin Curran, Christina Derksen, Dharani Yerrakalva, Daisy McInnerney, Paul M. Brennan, Fiona M. Walter, Suzanne E. Scott

**Affiliations:** 1 Centre for Cancer Screening, Prevention and Early Diagnosis, Wolfson Institute of Population Health, Queen Mary University of London, London, United Kingdom; 2 Department of Public Health and Primary Care, University of Cambridge, Cambridge, United Kingdom; 3 Translational Neurosurgery, Centre for Clinical Brain Sciences, University of Edinburgh, Edinburgh, United Kingdom; National Institutes of Health, University of the Philippines Manila / De La Salle University, PHILIPPINES

## Abstract

**Background:**

Brain tumours affect 7 per 100,000 people in the UK, glioma being most prevalent, with only 12% five-year survival rates and devastating impacts. Primary care triage tools could facilitate earlier detection of glioma. One option for triage is cognitive function testing. The aim of this systematic review was to determine if cognitive function tests can discriminate between patients with glioma and healthy controls, and their potential suitability for primary care use.

**Methods:**

Studies were included that conducted cognitive function tests with adult patients with glioma, prior to treatment, compared to healthy controls. Two independent researchers performed screening and data extraction. The primary outcome explored test discrimination between people with glioma and healthy controls.

**Results:**

Seventeen studies were identified. Findings indicated multiple cognitive function and language function have potential discriminatory capacity between patients with glioma and healthy controls. Over half of cognitive function tests measuring multiple cognitive functions (59%, n = 17) and language function (54%, n = 30) found significant differences between patients with glioma and healthy controls with medium or large effect size. The Montreal Cognitive Assessment has short test duration, high feasibility and acceptability, suggesting potential primary care suitability. Further acceptability and feasibility studies are needed for other potential tests.

**Conclusions:**

Acknowledging high heterogeneity of included studies, this review suggests tests of multiple cognitive functions or language could support primary care practitioners with decision-making for urgent neuroimaging referral. However, interpretations should be treated with caution and the applicability to primary care requires further exploration.

Prospero registration number: CRD42023408671

## Introduction

### Background

Brain tumours are experienced by approximately 7 per 100,000 in the UK population [1], have twelve-month survival rates of approximately 40% and five-year survival of only 12% [2]. Many patients (62%) are diagnosed through emergency services [3], which is associated with the poorest survival outcomes [4]. Even in patients who are referred through primary care, referral often only occurs after multiple visits to primary care, which adds to diagnostic delay. Over 40% of patients visit a GP more than three times before referral [5], which could be due to a lack of clear referral guidelines and diagnostic support tools [6]. This is also likely influenced by patients under-reporting their symptoms [7]. Research on detecting early symptoms is high priority for early detection of brain tumours [8], and is supported by the National Institute for Health and Care Excellence [9], the National Institute for Health and Care Research [10], the Cancer Research UK [11] research strategy, and the Tessa Jowell Brain Cancer Mission [12].

Only 1% of patients with brain tumours are referred via the urgent suspected cancer referral pathway [13], intended to expedite cancer diagnosis and reduce patient stress and anxiety [14]. Furthermore, GPs report that there are likely avoidable delays in the patient journey to diagnosis, especially in patients suffering from headaches without other symptoms [15]. Prompt diagnosis could facilitate treatment and survival outcomes with fewer treatment-associated deficits and reduced clinical deterioration [16,17]. Patient experience could also be improved by causing less patient stress and anxiety [18].

Identifying the wide spectrum of early symptoms is extremely challenging [4]. Most patients for whom imaging reveals a glioma present initially with seizures or neurological symptoms [19], including headache, cognitive changes, and non-specific symptoms [15,20]. The strongest positive predictive values for combined symptoms are headache with cognitive symptoms (7.2%) and cognitive symptoms alongside weakness (9.6%) [21]. Yet identifying which patients to refer for urgent neuroimaging is challenging [14], as headaches can signify more common conditions such as migraine [22], fatigue, muscular tension, anxiety and others, including no medical condition [23]. As brain tumours occur much less commonly than other conditions, GPs may first investigate other more common causes [4], which can add delays to the diagnostic pathway. It is also likely that patients do not interpret all cognitive changes as symptoms or disclose them to healthcare professionals [7]. Referring everyone with a headache would add unnecessary patient stress, overwhelm the already over-burdened neuroimaging service, and increase healthcare costs [13,24–26]. Identifying cognitive symptoms could help triage patients in primary care [27], alerting the potential need for prompt referral to neuroimaging [21,26]. A primary care triage support tool for cognitive symptoms could be a crucial innovation to both avoid over-referral and improve early detection rates [21]. However, tools are needed to identify cognitive deficits to assist GP referral decision-making [21].

Cognitive function tests identify neurological deficits through a series of tasks measured against validated cut-off scores [28]. In secondary care, cognitive function tests are used to monitor brain tumour progression, rehabilitation, and recurrence [29,30]. However, no gold-standard cognitive function test exists for patients with brain tumours [31]. Furthermore, testing for rehabilitation or surgical eligibility can be long and arduous (1–8 hours) for patients, thereby reducing clinical acceptability and feasibility [31], particularly within primary care. Evidence of the validity of short cognitive function tests for these patients is conflicting [30]. Therefore, it is crucial to identify whether individual cognitive function tests can identify cognitive impairments in patients with a brain tumour, while being acceptable and feasible in primary care considering resource, timing, and administration needs.

This systematic review aimed to determine if cognitive function tests can discriminate between patients with and without glioma prior to treatment. We chose to focus on glioma given that glioma has the highest incidence of all primary brain tumours [32] and rising prevalence [33]. Glioma represents over 80% of all malignant brain tumours [34]. Secondary aims were to determine details of what each test entails, how it was carried out, and any adverse effects experienced by participants to assess potential suitability for primary care.

## Methods

### Study design

This systematic review was carried out according to the Preferred Reporting Items for Systematic Review (PRISMA) guidelines [35]. The protocol was registered with the International Prospective Register of Systematic Reviews (PROSPERO) (CRD42023408671). The protocol included studies with patients with all brain tumour types; the results for glioma are presented here.

### Information sources

A search strategy (S1 Table) was conducted on 3^rd^ July 2024 to identify studies from the following electronic databases: Medline (via PubMed), CENTRAL, and Embase; citation searching of included studies; trials registers; and conference abstracts.

The full search strategy can be seen in the supplementary file (S1 Table). As an example, the following search strategy was used within the PubMed database: ((Brain tumour*[Title/Abstract]) OR (brain cancer*[Title/Abstract]) OR (brain neoplasms[MeSH Terms])) AND ((Cognit* function* test*[Title/Abstract]) OR (cognit* function* assessment*[Title/Abstract]) OR (cognitive function* exam*[Title/Abstract]) OR (executive function* test*[Title/Abstract]) OR (executive function* assessment*[Title/Abstract]) OR (executive function* exam*[Title/Abstract]) OR (neuropsycholog* assessment*[Title/Abstract]) OR (neuro-psycholog* assessment*[Title/Abstract]) OR (neuropsycholog* exam*[Title/Abstract]) OR (neuro-psycholog* exam*[Title/Abstract]) OR (cognit* test*[Title/Abstract]) OR (cognit* assessment*[Title/Abstract]) OR (cognit* exam*[Title/Abstract]) OR (cognit* abilit* test*[Title/Abstract]) OR (clock-drawing test[Title/Abstract]) OR (Montreal cognitive test[Title/Abstract]) OR (mini-mental state exam[Title/Abstract]) OR (abbreviated mental test[Title/Abstract]) OR (memory impairment screen[Title/Abstract]) OR (mental status questionnaire[Title/Abstract]) OR (short portable mental status questionnaire[Title/Abstract]) OR (neuropsychiatric inventory questionnaire[Title/Abstract]) OR (mini examen cognoscitivo[Title/Abstract]) OR (Eurotest[Title/Abstract]) OR (Fototest[Title/Abstract]) OR (memory alteration test[Title/Abstract]) OR (verbal fluency[Title/Abstract]) OR (memory[Title/Abstract]) OR (mental capacity[Title/Abstract]) OR (Neuropsychological tests[MeSH Terms])).

### Eligibility criteria

Studies were included that used an objective cognitive function test with adult patients with primary glioma from any gender, ethnicity, or socioeconomic background and a control group that was either healthy or with any condition other than brain tumour. All study designs with a control group were included. Studies were included in all languages, timeframes, and publication statuses.

Studies were excluded if participants completed cognitive testing after treatment or biopsy as this could impact cognitive function [36]. Studies were excluded if patients had a metastatic tumour diagnosis, or if there was no control group. Case studies with <5 patients or controls were also excluded. One study [37] that included a control group with hippocampal sclerosis was excluded from the review as it was not comparable to the other studies in which control groups were without neurological conditions. This study also had a high risk of bias.

### Study selection

Two researchers (LS, and a member of the reviewer team (RC, AA, SB, TM, DY, DM, JL, CD, PA, SA)) independently screened all abstracts and full texts to assess their eligibility for the review. Any disagreements were taken to a third researcher for decision.

### Data extraction and management

Data were extracted independently by two researchers (LS and either SS, RC, RE or CD), including participant demographics; recruitment methods; tumour type, stage, and location; cognitive function tests and administration; study design and methods; study completion rates, outcomes and results; and author conflicts of interest, where relevant. Details of cognitive function test administration (i.e. duration, timepoint, setting, mode of delivery, and administered by whom) were used to consider suitability for use in primary care.

Where data was missing, incomplete, or unclear, authors were contacted for clarification where possible. Missing data were reported as no data reported (n.d.). Quality assessment conducted during data extraction was based on GRADE and QUADAS-2 for diagnostic accuracy studies [38], assessing patient selection, testing, flow and timing, selective outcome reporting, and measurement of outcomes. A study was assessed as having overall low risk of bias if all categories were deemed to be low risk. A study was assessed as having overall moderate risk of bias if one or more (but not all) categories were found to have moderate risk. A study was assessed as having a high risk of bias if at least one category was high risk or all categories were moderate risk [39]. As a meta-analysis was not carried out, other factors contributing to GRADE certainty of evidence were assessed narratively. Inconsistency was assessed using effect sizes and imprecision was assessed using sample sizes, compared across studies were relevant and appropriate. Indirectness was assessed using the relevance of the studies to the review research questions, and publication bias was assessed using selective reporting and declared funding sources.

During the data extraction process, it became apparent that there was variation in the claimed cognitive functions being assessed by each cognitive test, and inconsistencies in definitions of cognitive functions between studies. In particular, executive function has been previously defined to include inhibition and interference control, working memory, and cognitive flexibility [40], and as a wider concept encompassing multiple cognitive functions [41]. To guide synthesis and interpretation in this review, each cognitive test was categorised as measuring one of seven cognitive functions (language, memory, information processing, executive function, decision-making, attention, and visuospatial function) or multiple cognitive functions. All cognitive function tests used across all included studies were listed alongside the cognitive function(s) each test was measuring, as reported by each study. This was compared across all included studies to ensure consistency. Existing literature using the same tests was consulted where there was uncertainty or conflicts. Where there was conflict in the claimed measured cognitive function, the majority consensus was adopted and validated using existing literature. (S2 File).

Effect sizes for test findings were calculated using Hedges’ *g*, with small effect 0.2, medium effect 0.5, and large effect 0.8 [42]. Subgroup analysis of frontal and temporal lobe tumours was included as this tumour location is most likely to cause cognitive symptoms.

## Results

**Fig 1:**
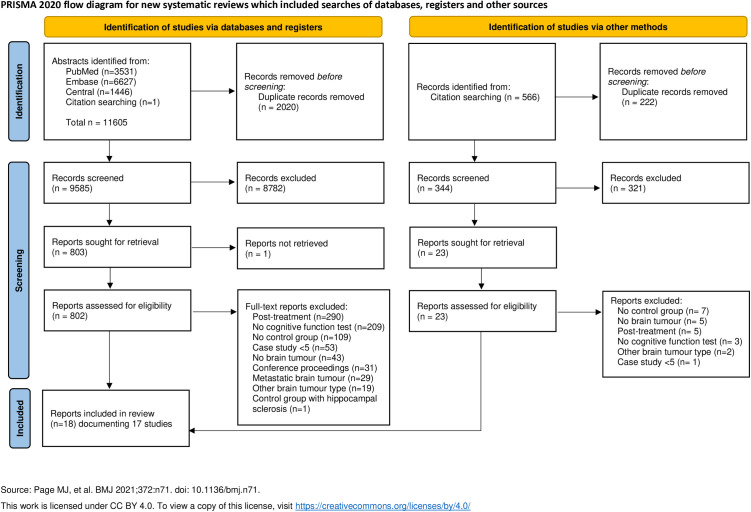
PRISMA flow diagram.

A total of 11,605 records were identified by the search. After duplicate removal, 9,585 abstracts were screened, and 803 full texts were assessed for inclusion. One full text [43] was not able to be retrieved. Eighteen records met the inclusion criteria and were included in this review: two reported the same longitudinal study [44,45]. See Fig 1 for the PRISMA flow diagram.

### Study characteristics

Table 1 presents summary data for all included studies (n = 17) [44–61]. Further study characteristics are presented in S3 Table, S4 Table, and S5 Table.

**Table 1:** Summary of studies included in systematic review.

Study	Patients	Controls	Cognitive Testing									
			Duration (minutes)	Timepoint for patient testing	Setting/ Mode of Delivery	Administered By	Cognitive Function	Measure	Mean (SD)		Significance Testing	Effect size
	N [Tumour type N] (location %)	Condition N							Patients	Controls		(Hedges’ *g*)
Reijneveld et al 2001 [46]	24 [Astrocytoma 21 Missing data 3] (n.s.^a^)	Healthy 23	90	Diagnosis to test interval mean 4.4 years (4.5)	Fixed order of tests	Trained psycho-metricians supervised by a neuro-psychologist	Multiple cognitive functions	LDMT^*^	^†^	z = 0	n.d.	–
								Stroop^§^	^†^	z = 0	n.d.	–
							Memory	VVLT^*^	^†^	z = 0	n.d.	–
								WAIS^*^^,^^§^ Working Memory	^†^	z = 0	n.d.	–
							Language	VFT^*^ Semantic	^†^	n.d.	n.d.	–
							Executive function	Concept Shifting Test	^†^	z = 0	n.d.	–
Ruge et al 2010 [47]	33 [Astrocytoma 30 Oligodendroglioma 2 Oligoastrocytoma 1] (Left 55% Right 45% Frontal 33% Temporal/Insular 55% Parietal 6% Subcortical 6%)	Healthy 33	90–150	14 days before surgery or biopsy	Department of Physical Medicine and Rehab-ilitation at the Ludwig-Maximilians University	n.d.	Memory	MVGT^*^ Learning Trial 1–5	47.5 (7)	49.3 (7.5)	p > .4	−0.25
								MVGT^*^ List 2	5.7 (1.3)	5.8 (1.7)	p > .56	−0.07
								MVGT^*^ Free Recall 1	9 (2.7)	9.2 (2.8)	p > .82	−0.07
								MVGT^*^ Free Recall 2	9.1 (2.5)	9.9 (2.9)	p > .26	−0.29
								MVGT^*^ Distraction 1	−3.3 (1.9)	−2.8 (1.8)	p > .27	−0.27
								MVGT^*^ Distraction 2	0.09 (1.5)	0.7 (1.3)	p > .08	−0.43
Bizzi et al 2012 [48]	19 [Oligodendroglioma 3 Astrocytoma 2 Oligoastrocytoma 3 Glioblastoma multiforme 11] (Left frontal 100% VLPM^b^ 42% VLPC^b^ 58%)	Healthy 10	n.d.	Within one week before surgery	n.d.	Two neuro-psychologists with 8 and 16 years of experience	Language	AAT^*^ Written Language	VLPM: 88.9 (1.4) VLPC: 81.0 (16.0)	90 (0)	VLPM: p = .083 VLPC: p = .007	VLPM: −1.09 VLPC: −0.76
								AAT^*^ Comprehension	VLPM: 113.2 (11.1) VLPC: 114.6 (5.0)	119.0 (1.3)	VLPM: p = .203 VLPC: p = .011	VLPM: −0.72 VLPC: −1.15
								AAT^*^ Communicative Behaviour	VLPM: 4.9 (.4) VLPC: 4.1 (1.1)	5 (0)	VLPM: p = .696 VLPC: p = .021	VLPM: −0.35 VLPC: −1.11
								AAT^*^ Articulation and Prosody	VLPM: 4.9 (.4) VLPC: 3.9 (.9)	5 (0)	VLPM: p = .694 VLPC: p = .002	VLPM: −0.35 VLPC: −1.66
								AAT^*^ Phonological Structure	VLPM: 4.9 (.4) VLPC: 4.2 (.9)	5 (0)	VLPM: p = .696 VLPC: p = .021	VLPM: −0.35 VLPC: −1.20
								Token Test	VLPM: 45.5 (10.4) VLPC: 44.5 (5.6)	49.1 (1)	VLPM: p = .762 VLPC: p = .004	VLPM: −0.48 VLPC: −1.10
								VFT^*^ Semantic	VLPM: 39.6 (12.1) VLPC: 33.3 (13.7)	50.7 (9.4)	VLPM: p = .083 VLPC: p = .002	VLPM: −0.99 VLPC: −1.42
								VFT^*^ Phonemic	VLPM: 32.5 (10.6) VLPC: 18.5 (10.9)	44.7 (11.3)	VLPM: p = .055 VLPC: p = .000	VLPM: −1.06 VLPC: −2.26
Mattavelli et al 2012 [49]	22 [Oligodendroglioma 17 Glioblastoma 2 Astrocytoma 3] (Left frontal 100%)	Healthy 26	n.d.	In the month before surgery	Computer – E-prime experi-mental software	n.d.	Decision-making	Gambling Task: Good	27.72 (13.69)	43 (22.74)	p = .008	−0.78
								Gambling Task: Bad 1	33.32 (13.07)	30.46 (17.31)	p = .53	0.18
								Gambling Task: Neutral	20.95 (7.94)	13.23 (7.15)	p = .001	1.01
								Gambling Task: Bad 2	18 (6.71)	13.30 (6.5)	p = .018	0.70
								Gambling Task: Reaction Time	n.d.	n.d.	p = .003	–
Mu et al 2012 [50]	11 [Astrocytoma 4 Glioblastoma multiforme 2 Anaplastic astrocytoma 3 Oligodendroglioma 2] (Left frontal 100%)	Healthy 11	90	3–5 days prior to surgery	Relatively quiet environment with bright lighting, and comfortable chairs and desks	n.d.	Multiple cognitive functions	DST^*^ Total	10.18 (2.316)	13.91 (2.587)	p = .000	−1.46
								DST^*^ Forwards	6.36 (1.567)	8.18 (1.079)	p = .016	−1.30
								DST^*^ Backwards	3.91 (1.375)	5.73 (1.954)	p = .003	−1.04
							Memory	Tapping Test Total	14.64 (2.501)	16.82 (2.04)	p = .079	−0.92
								Tapping Test Forwards	7.73 (1.348)	9 (1.265)	p = .067	−0.93
								Tapping Test Backwards	6.91 (1.64)	7.82 (1.25)	p = .194	−0.60
							Executive function	Modified Card Sorting Test: Category Control	3.27 (1.737)	4.27 (.905)	p = .128	−0.69
								Modified Card Sorting Test: Preservative Response	6.18 (4.119)	2.73 (3.101)	p = .047	0.91
								Modified Card Sorting Test: Failure to Maintain a Set	1.55 (1.036)	1.27 (1.489)	p = .615	0.21
								Modified Card Sorting Test: Preservative Error	1.82 (1.537)	0.73 (.905)	p = .082	0.83
								Modified Card Sorting Test: Number of Answers to Complete First Category	11.91 (10.58)	9.18 (3.188)	p = .574	0.34
								Modified Card Sorting Test: % Preservative Response	40.91 (21.72)	53.41 (11.31)	p = .128	−0.69
								Modified Card Sorting Test: Total Errors	18.73 (6.405)	14.82 (3.763)	p = .061	0.72
Plaza et al 2013 [51]	10 [Oligoastrocytoma 5 Astrocytoma 1 Oligodendrioma 4] (Right fronto-temporo-parietal 10% Right front-temporo-insular 30% Left temporal 10% Left fronto-temporo-basal 10% Right temporo-insular 10% Left frontal 10% Left fronto-callo-parieto-cingular 10% Right parietal 10%)	Healthy Paris community members and patients’ relatives 60	45	Pre-surgery	Participants seated in front of 17-inch computer at viewing distance of approx. 70 cm; task order counter-balanced across participants; preceded by a training phase	Neuro-psychologist oversaw protocol	Multiple cognitive functions	Dual-Attention Task Visual	49.9 (12.5)	69.8 (6.8)	p < .001	−2.52
								Dual-Attention Task Auditory	5.8 (1.3)	6 (1.4)	p = n.s.	−0.14
							Information processing	Learning-Meaningful Tasks: Visual	62 (2.9)	62.8 (1.5)	p = n.s.	−0.45
								Learning-Meaningful Tasks: Auditory	63.9 (.3)	62.8 (1.5)	p = n.s.	0.78
								Learning-Meaningful Tasks: Crossmodal	63.2 (1.3)	63.1 (1.3)	p = n.s.	0.15
								Learning-Non-Meaningful Tasks: Visual	60.3 (4.5)	61.9 (1.9)	p = n.s.	−0.66
								Learning-Non-Meaningful Tasks: Auditory	63.5 (1)	63.6 (.6)	p = n.s.	−0.15
								Learning-Non-Meaningful Tasks: Crossmodal	61.9 (2.4)	62.4 (1.4)	p = n.s.	−0.32
								Matching Tasks: Visual	22.5 (1.2)	22.7 (1.1)	p = n.s.	−0.18
								Matching Tasks: Auditory	22.1 (1.5)	23 (1.1)	p < .05	−0.77
								Matching Tasks: Crossmodal	68.8 (.1)	69 (.9)	p = n.s.	−0.24
								Picture Naming Task: Score	68.8 (.7)	69.6 (.6)	p < .01	−1.29
								Picture Naming Task: Time (s)	145.1 (28.2)	111.6 (16.6)	p < .001	1.79
Satoer et al 2013 [44] *and* 2018 [45]	27 a*nd* 18 [Glioma 27 *and* 18] (Left (n.d.) Language area 56% Non-language area 37% Unknown 8%) *and* (Left 89% Language area 61% Non-language area 28% Unknown 11%)	Healthy 21	n.d.	1 month pre-operatively	Patients: Interview setting Controls: private setting (home or other quiet environ-ment) 300 words selected per participant for linguistic analysis	n.d.	Language	BNT^*^	n.d.	n.d.	p = .001	–
								Spontaneous Speech: Lexical Diversity	0.46 (.04)	0.48 (0.3)	p = .184	−0.10
								Spontaneous Speech: MLUw	8.74 (1.67)	9.5 (2.05)	p = .0168^‡^	−0.41
								Spontaneous Speech: Repetitions	10.70 (9.63)	6.05 (5.44)	p = .055	0.57
								Spontaneous Speech: Self-Corrections	5.65 (4.83)	3.86 (2.17)	p = .122	0.45
								Spontaneous Speech: Incomplete Sentences	7.8 (6.79)	2 (2.34)	p = .001	1.07
								VFT^*^ Semantic	n.d.	n.d.	p < .001	–
Habets et al 2014 [52]	62 [Glioblastoma multiforme 57 Astrocytoma oligoastrocytoma 5] (Left 45% Right 55%)	Healthy n.d.	n.d.	In the week preceding surgery (mean 4 days (7.9) median 1, range 1–49	n.d.	n.d.	Multiple cognitive functions	LDMT^*^	^†^	z = 0	p < .001	–
								Stroop^§^	^†^	z = 0	p < .001	–
							Memory	VVLT^*^	^†^	z = 0	p < .001	–
							Executive function	Concept Shifting Test	^†^	z = 0	p < .001	–
Huang et al 2014 [53]	12 [Glioma 12] (Left 58% Right 42%)	Healthy hospital staff 12	n.d.	Before operation	n.d.	Professional appraiser in the neuro-psychological research centre	Multiple cognitive functions	MoCA^*^	20.2 (1.5)	27.9 (1.1)	p < .01	−5.65
Kinno et al 2014 [54]	21 [Anaplastic astrocytoma 1 Anaplastic oligodendroglioma 6 Anaplastic oligoastrocytoma 4 Diffuse astrocytoma 5 Oligoastrocytoma 3 Oligodendroglioma 2] (Left 100% LPMC^c^ 33% F3^c^ 33% Other left frontal regions 33%)	Healthy 28	n.d.	n.d.	n.d.	n.d.	Language	Picture-Sentence Matching Task: Error Rates Active	8.13	3.1	p < .0001	–
								Picture-Sentence Matching Task: Error Rates Passive	11.82	2.9	p < .0001	–
								Picture-Sentence Matching Task: Error Rates Scrambled	22.34	2.2	p < .0001	–
								Picture-Sentence Matching Task: Error Rates One-Argument	2.4	1.8	p = .76	–
								Picture-Sentence Matching Task: Error Rates Control Test	2.32	2.2	n.d.	–
								Picture-Sentence Matching Task: Reaction Times (ms) Active	3334	2052	n.d.	–
								Picture-Sentence Matching Task: Reaction Times (ms) Passive	3399	3129	n.d.	–
								Picture-Sentence Matching Task: Reaction Times (ms) Scrambled	3499	3242	n.d.	–
								Picture-Sentence Matching Task: Reaction Times (ms) One-Argument	2760	2644	n.d.	–
								Picture-Sentence Matching Task: Reaction Times (ms) Control Test	2756	2766	n.d.	–
Antonsson et al 2018 [55]	23 [Oligoastrocytoma 5 Astrocytoma 7 Glioblastoma multiforme 2 Oligodendroglioma 6 Ganglioglioma 3] (Left 70% Right 30%)	Healthy 80	120–180	mean 14 days before surgery range 1–72 days	Patients in a hospital setting; control group not specified; video-recorded for double checking; BNT computer-ised; two sessions with long break in between	Patients: first author Controls: 5 final-year students from speech and pathology programme at University of Gothenburg	Language	BeSS^*^ Total	181.2 (18.1)	180.6 (16.27)	p = .745	0.04
								BeSS^*^ Repetition	20.9 (5.33)	21.8 (4.99)	p = .524	−0.18
								BeSS^*^ Recreating Sentences	26 (3.91)	25.3 (3.41)	p = .267	0.20
								BeSS^*^ Making Inferences	27.9 (2.56)	27.4 (2.52)	p = .273	0.20
								BeSS^*^ Comprehension Logico-Grammatical	26.3 (3.2)	27.3 (3.52)	p = .122	−0.29
								BeSS^*^ Comprehension Ambiguous	25.1 (4.96)	25.9 (4.31)	p = .567	−0.18
								BeSS^*^ Metaphors	28.3 (2.15)	26.6 (3.27)	p = .014	0.55
								BeSS^*^ Word Definitions	26.6 (4.24)	26.5 (2.89)	p = .239	0.03
								BNT^*^	50.9 (5.24)	53.9 (3.62)	p = .034	−0.74
								Spontaneous Speech: Sentence Analysis	50.2 (6.74)	50.6 (4.98)	p = .827	−0.07
								Spontaneous Speech: Morphological Completion	42.1 (4.08)	42.5 (4.73)	p = .451	−0.08
								VFT^*^ Semantic	Animals: 22.4 (5.33) Verbs: 19 (6)	Animals: 25.4 (5.31) Verbs: 21.4 (6.33)	Animals: p = .038 Verbs: p = .066	Animals: −0.56 Verbs: −0.38
								VFT^*^ Phonemic	43 (12.7)	45.5 (10.6)	p = .345	−0.22
De Witte et al 2018 [56]	14 [Glioblastoma 8 Oligodendroglioma 6] (Left 100% Frontal 29% Temporal 36% Insular 7% Temporal or insular 14% Parietal 14%)	Healthy 14	≤20	Before surgery	Sit alone and turn off all media; telephone; 7 patients in A-B-A-B order; 7 patients in B-A-B-A order (same for controls)	Speech language pathologists and neuro-psychologists trained in language assessments	Language	TeleLanguage Test: Verbal Naming Task	n.d.	n.d.	p = .001	–
								TeleLanguage Test: Letter fluency	n.d.	n.d.	p = .008	–
								TeleLanguage Test: Semantic Fluency	n.d.	n.d.	p = .001	–
Zhang et al 2018 [57]	78 [Glioma 78] (Left 100%)	Healthy volunteers from advertisement 44	n.d.	n.d.	n.d.	Neuro-psychologist	Language	ABC^*^ Naming	90.86 (9.69)	97.36 (3.05)	p < .001	−0.81
								ABC^*^ Repetition	94.88 (8.86)	99.79 (.59)	p < .001	−0.69
								ABC^*^ Comprehension	215.71 (21.04)	227.25 (4.5)	p < .001	−0.67
								ABC^*^ Spontaneous Speech	18.46 (1.88)	19.84 (.57)	p < .001	−0.89
								AQ^*^	92.82 (7.45)	98.93 (1.69)	p < .001	−1.00
								BNT^*^	20.69 (5.17)	27.93 (2.5)	p < .001	−1.63
							Executive function	MMSE^*^	Median 27	Median 30	p < .001	–
Hu et al 2020 [58]	17 [Glioma 17] (Left 47% Right 53% Temporal lobe 100%)	Healthy 28	n.d.	n.d.	n.d.	Neuro-psychologists	Multiple cognitive functions	DST^*^ Total	8.44 (3.09)	11 (2.67)	p = .518	−0.89
								DSST^*^	7.4 (2.88)	11.88 (1.64)	p = .028	−2.01
								Arithmetic ^§^	5.89 (2.42)	10.63 (2)	p = .007	−2.15
							Memory	Memory test^§^	6.43 (4.72)	11.88 (1.55)	p = .063	−1.71
							Language	WAIS^*^ Similarities Test	6.38 (2.88)	10 (1.07)	p = .035	−1.82
							Visuospatial function	Mapping^§^	5.63 (2.56)	9.88 (.64)	p = .000	−2.54
								Visuospatial test ^§^	7 (3.87)	10.63 (1.6)	p = .021	−1.33
Mooijman et al 2022 [59]	36 [Astrocytoma 13 Oligodendroglioma 12 Glioblastoma 10 Xanthoastrocytoma 1] (Left 67% Right 33% Frontal 53% Temporal 19% Insular 3% Parietal 8% Frontoparietal 5% Parietotemporal 3% Temporoparietal 3% Frontotemporal 5.5%)	Healthy 35	15	Pre-operative	Controls tested in a private setting; standard clinical work-up; random order of tests	Clinical staff	Multiple cognitive functions	TMT^*^ A	29.61	n.d.	p = .17	–
								TMT^*^ B	79.39	n.d.	p = .03	–
								TMT^*^ BA	2.60	n.d.	p = .04	–
							Language	BNT^*^	48.88	52.9	p = .03	–
								Token Test	33.86	n.d.	n.d.	–
							Information processing	Sentence Judgement Test: Time (ms)	n.d.	n.d.	p = .84	–
								Sentence Judgement Test: Semantics (ms)	2810.29	n.d.	n.d.	–
								Sentence Judgement Test: Syntax (ms)	3251.90	n.d.	n.d.	–
								Sentence Judgement Test: Phonology (ms)	2234.57	n.d.	n.d.	–
								Sentence Judgement Test: Accuracy	13.57	n.d.	p = .01	–
Tarantino et al 2022 [60]	22 [Glioma 22] (Left 50% Right 36% Bilateral 14% Frontal 36% Temporal 18% Parietal 13% Anterior cingulate 5% Insula 5% Multiple 23%)	Healthy 20	n.d.	n.d.	Paper and pencil and two tasks on computer (AX-CPT and Stroop)	n.d.	Multiple cognitive functions	DST^*^ Forwards	5.1 (1)	5.5 (.8)	p = .125	−0.43
								DST^*^ Backwards	3.6 (1.7)	4.3 (1.1)	p = .299	−0.47
								TMT^*^ A	34.2 (11.3)	33.6 (9.9)	p = .896	0.06
								TMT^*^ B	131.6 (78.6)	103.2 (50.1)	p = .081	0.42
								Stroop^§^ Response Accuracy	n.d.	n.d.	p = .822	–
								Stroop^§^ Trial Order Accuracy	n.d.	n.d.	p = .407	–
								Stroop^§^ Response Time	n.d.	n.d.	p = .241	–
								Stroop^§^ Trial Order Time	n.d.	n.d.	p < .001	–
							Memory	Corsi Forwards	5.2 (1)	5.7 (1.1)	p = .219	−0.47
								Corsi Backwards	4.5 (1.1)	4.9 (1.1)	p = .274	−0.36
								Memory with Interference (10s)	6.6 (2)	6.6 (2.5)	p = .922	0
								Memory with Interference (30s)	5 (2.4)	6.6 (2.7)	p = .021	−0.62
								Story Recall Immediate	9.9 (3.4)	14.2 (4.4)	p = .002	−1.08
								Story Recall Delayed	12 (4.4)	17.2 (3.8)	p = .001	−1.24
							Language	BNT^*^	13.2 (1.7)	14.2 (.9)	p = .016	−0.71
								VFT^*^ Phonemic	10.9 (4.3)	13.9 (7.3)	p = .095	−0.50
							Attention	CPT^*^ Response Accuracy	n.d.	n.d.	p = .33	–
								CPT^*^ Trial Order Accuracy	n.d.	n.d.	p = .002	–
								CPT^*^ Response Time	n.d.	n.d.	p = .225	–
								CPT^*^ Trial Order Time	n.d.	n.d.	p < .001	–
Wang et al 2022 [61]	90 [Glioma 90] (Left 50% Right 48% Bilateral 2%)	Healthy volunteers 50	n.d.	1–3 days before surgery	n.d.	Researchers trained in neuro-psychological assessments	Multiple cognitive functions	SDMT^*^	38.4 (15.4)	48.9 (18.3)	p = .000	−0.63
								TMT^*^ A	68.5 (32.6)	49.7 (24.5)	p = .000	0.62
								TMT^*^ BA	89.6 (74.0)	67.1 (41.4)	p = .038	0.35
								Stroop^§^ B	47.8 (26.8)	34.9 (8.1)	p = .000	0.58
								Stroop^§^ CB	44.9 (27.7)	28.4 (11.1)	p = .002	0.71
							Memory	AVLT^*^	5.5 (2.8)	7.6 (2.2)	p = .000	−0.80
							Language	BNT^*^	23.2 (3.9)	25.6 (3.0)	p = .000	−0.66
								VFT^*^ Semantic	18.2 (5.6)	25.4 (5.9)	p = .000	−1.25
							Executive function	MMSE^*^	27.8 (2)	28.9 (1.3)	p = .000	−0.61

^a^Demographics reported for full sample only

^b^Anterior ventrolateral premotor (VLPM); posterior ventrolateral precentral (VLPC)

^c^Left lateral premotor cortex (LPMC); opercular/triangular parts of the left F3 (F3)

* AMIPB: Adult Memory and Information Processing Battery; AVLT: Auditory Verbal Learning Test; DST: Digit Span Test; MoCA: Montreal Cognitive Assessment; MVGT: Munchner Verbaler Gedachtnistest; VVLT: Visual Verbal Learning Test; WAIS: Wechsler Adult Intelligence Scale; AAT: Aachener Aphasie Test; ABC: Aphasia Battery for Chinese Speakers; AQ: Aphasia Quotient; BeSS: Bedomning av subtila sprakstorningar; BNT: Boston Naming Test; CPT: Conner’s Continuous Performance Test; DSST: Digit-Symbol Substitution Test; LDMT: Letter Digit Modalities Test; MMSE: Mini Mental State Examination; SDMT: Symbol-Digit Modalities Test; TMT: Trail-Making Test;

† Graphical presentation of data only

‡ Not significant as defined by authors of included study

§ Specific test not specified; stated as reported in included study

n.s. Not specified

n.d. No data reported

Nearly all studies (n = 16) [44,45,47–61] reported tumour location. Studies were typically carried out in mainland Europe (n = 10) [44–49,51,52,55,59,60] or China (n = 5) [50,53,57,58,61]. Three studies [46,51,55] reported study design as case-control. Other study designs were reported as prospective [47,48,52,61], observational [44,45,61], exploratory [55,56], or longitudinal [44,45]. Three-quarters of studies (n = 13) [47,49–55,57–61] were single-centre. Study aims explored cognitive function status (n = 10) [44–47,49,50,52,55,58,60,61]; anatomical / topological mapping (n = 7) [48,51,53,54,57,58,60]; treatment-associated cognitive impairment (n = 5) [44–46,52,53,61]; and test validation (n = 2) [49,56]. Only two studies [46,51] specifically aimed to explore cognitive discrimination between patients with and without glioma.

Patients were recruited from medical settings (n = 14) [46,47,49–53,55–61], often as in-patients for neurosurgery (n = 8) [47,49–51,55,57,58,60]. Control groups were healthy participants (n = 17) [44–61], typically volunteers (n = 6) [46,47,51,55,57,61], patients’ family members [51] or research / hospital staff [53]. Most studies (n = 10) [44,45,48–50,52,54,56,58–60] did not report the method of recruitment of controls.

Most studies (n = 15) [44–46,48–50,52–61] reported inclusion and exclusion criteria for patients. Fewer studies (n = 9) [44,45,48,49,55–60] reported these for controls. Many studies excluded participants with severe cognitive deficits [44–46,48,50,52,54–57,60], comorbidities [44,45,48,50,52–61] or psychiatric illness [44,45,48,50,54,56–60], drug, alcohol, or substance abuse [44,45,48,56–59], or medication affecting cognitive function [46,56,59]. Some studies included only patients eligible for surgery [48,50,52,56,59]. Some studies excluded older age patients [44,45,50,57,60].

The average number of patients in each study was n = 30 (range 10–90). The average number of controls was n = 31 (range 10–80). Studies excluded patients from data analysis for medical complications (n = 3) [52,55,59], incompatible histological diagnosis (n = 3) [47,55,61], fatigue or cognitive impairment (n = 2) [52,60], or organisational reasons (n = 1) [52]. In three studies [46,58,59] there was missing test data without reason.

Participant average ages ranged from 31.8 to 60.6 years old. Thirteen studies [44,45,48–53,55–57,59–61] reported patient education levels. No studies reported ethnicity or socioeconomic status. Many studies matched controls for age (n = 14) [44–47,49–53,55,56,58–61], education (n = 12) [44–50,52,53,55,56,59,60], and sex (n = 10) [46,47,50–53,56,58,60,61]. Some studies reported whether patients (n = 7) [46,47,50,52,54,55,61] or controls [61] had any symptoms or signs such as seizures [46,47,50,52,54,55], headache [47,50,52], or neurological deficits [52], and medication influencing cognitive function [46,47,50,52]. Some studies tested handedness (n = 12) [44–46,50,51,53–60] (matched to controls [44–46,50,51,53,57,58,60]), functional impairment [46,47,57,61], intelligence (matched to controls [50]), or anxiety and depression (matched to controls and controlled for as covariates [50]). Most studies did not report controlling for these factors when making comparisons.

The average number of cognitive function tests administered was 6 (range 1–14). Test duration was reported in seven studies [46,47,50,51,55,56,59], and ranged from 15–180 minutes. There was wide variation in test duration: one study [59] reported 15-minute duration for a total of six cognitive tests, and another [56] reported ≤20-minute duration for one language assessment. Six studies [44,45,47,50,55,56,59] reported the physical test location (typically a quiet environment with breaks between tests), while six studies [44,45,49,51,55,56,60] reported the mode (e.g. computerised). In ten studies [46,48,51,53,55–59,61] expert professionals administered testing, while seven studies [44,45,47,49,50,52,54,60] did not specify the administrator. Only four studies stated whether test order was counterbalanced [51,56], randomised [59] or fixed [46]. All studies performed cognitive function testing prior to treatment, including any tumour-directed therapy, biopsy, or peri-operative steroid use; however, four studies [54,57,58,60] did not specify precisely how long before treatment the cognitive function tests were administered. The specific timepoints reported for testing ranged from 1 day to 1 month before surgery; however, other less specific timepoints were also reported (e.g. “before surgery” [56]). Only two studies reported the timepoint of testing for controls, being the same time as patients [50] or from a previous study [54].

### Quality and risk of bias

**Fig 2:**
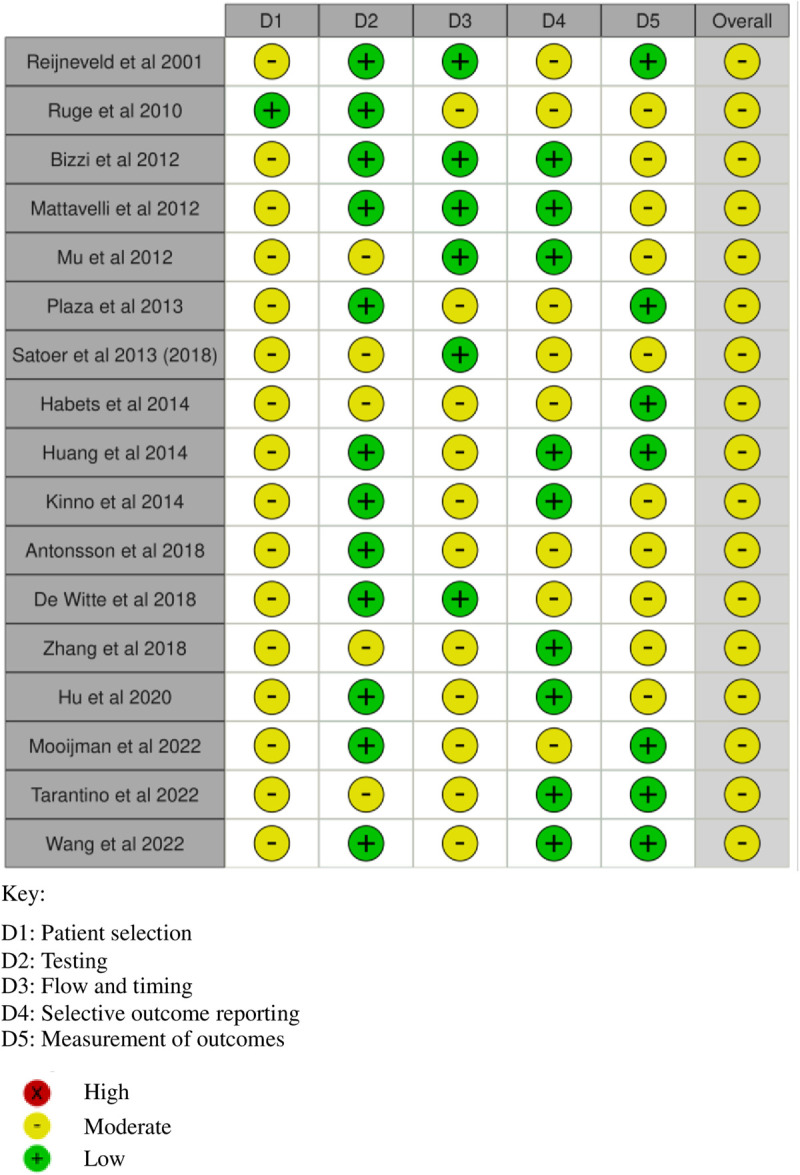
Risk of bias plot for studies including patients with glioma.

In all studies [44–61] there was risk of bias. All studies, except one [47], had moderate risk of bias over patient selection, typically excluding patients with severe cognitive deficits. Five studies [44,45,50,52,57,60] had moderate risk of bias for testing, typically not specifying blinding or test administration [43–45,47–49,55]. Eleven studies [47,51–55,57–61] had moderate risk of bias for flow and timing, typically not specifying test procedure [47–49,52,53,56,57,59,60]. Half of studies (n = 8) [44–47,51,52,55,56,59] had moderate risk of bias for selective outcome reporting, typically for not reporting non-significant values [45,50–52,58–60]. Ten studies [44,45,47–50,54–58] had moderate risk of bias for outcome measurement, typically due to wide confidence intervals. (Fig 2).

### Findings

Tables 1 and 2 summarise the findings of the included studies.

**Table 2:** Matrix showing which cognitive function was tested in which studies and whether a significant difference was found between patients and control groups.

Study	Control Group Condition	Cognitive Function							
GLIOMA		Multiple Cognitive Functions	Language	Memory	Information Processing	Executive Function	Decision-Making	Attention	Visuospatial Function
Reijneveld et al 2001 [46]	Healthy	••	•	••		•			
Ruge et al 2010 [47]	Healthy			••••••					
Bizzi et al 2012 [48]	Healthy		↓↓↓↓↓↓↓↓						
Mattavelli et al 2012 [49]	Healthy						↓↓↓↓ •		
Mu et al 2012 [50]	Healthy	↓↓↓		•••		↓ ••••••			
Plaza et al 2013 [51]	Healthy	↓ •			↓↓↓ ••••••••				
Satoer et al 2013 [44] *and* 2018 [45]	Healthy		↓↓↓ ••••						
Habets et al 2014 [52]	Healthy	↓↓		↓		↓			
Huang et al 2014 [53]	Healthy	↓							
Kinno et al 2014 [54]	Healthy		↓↓↓ • °°°°°°						
Antonsson et al 2018 [55]	Healthy		↓↓↑ •••••••••••						
De Witte et al 2019 [56]	Healthy		↓↓↓						
Zhang et al 2018 [57]	Healthy		↓↓↓↓↓↓			↓			
Hu et al 2020 [58]	Healthy	↓↓ •	↓	•					↓↓
Mooijman et al 2022 [59]	Healthy	↓↓ •	↓ •		↓ • °°°				
Tarantino et al 2022 [60]	Healthy	↓ •••••••	↓ •	↓↓↓ •••				↓↓ ••	
Wang et al 2022 [61]	Healthy	↓↓↓↓↓	↓↓	↓		↓			
Note: Each bullet point or arrow represents one test measured in the cognitive domain that is listed.									
**Key**									
↓	Patients performed significantly worse than controls								
↑	Patients performed significantly better than controls								
•	No significant difference between patients and controls								
°	No significance testing performed								

#### Multiple cognitive functions.

Nine studies [46,50–53,58–61] explored tests measuring multiple cognitive functions, reporting twenty-nine test results. Seventeen test findings across eight studies [50–53,58–61] found patients to have significantly worse cognitive function than healthy controls, reporting mostly medium and large effect sizes (using Digit Span Test (DST), Dual-Attention Task Visual, Letter Digit Modalities Test, Stroop, Montreal Cognitive Assessment (MoCA), Digit Symbol Substitution Test (DSST), Arithmetic, Trail-Making Test (TMT), and Symbol Digit Modalities Test). Effect size was not calculable for six findings [52,59,60] as patient and control scores were not reported. Twelve test findings across five studies [46,51,58–60] found no significant differences in cognitive function between patients and healthy controls (using Letter Digit Modalities Test, Stroop, Dual Attention Task Auditory, DST, and TMT). Six non-significant findings [51,60] showed a small or medium effect. One non-significant finding [58] showed a large negative effect, where patients performed worse than healthy controls.

Sub-group analysis of tumour location showed three studies [50,53,58] explored tests measuring multiple cognitive functions in patients with only frontal or temporal lobe glioma, reporting seven test findings. Six of the seven test findings [50,53,58] demonstrated patients performed significantly worse than healthy controls. In the remaining test finding [58] cognitive function did not differ between patients and controls.

#### Language.

Eleven studies [44–46,48,54–61] explored language as a cognitive function, reporting fifty-six test results. Thirty test findings across ten studies [44,45,48,54–61] found patients to have significantly worse language than healthy controls, nearly all with medium or large effect (using Aachener Aphasie Test, Aphasia Battery for Chinese Speakers, Aphasia Quotient, Boston Naming Test, Picture-Sentence Matching Task, Spontaneous Speech, TeleLanguage Test, Token Test, Verbal Fluency Test, and Wechsler Adult Intelligence Scale Similarities). Effect size was not calculable for nine findings [44,45,54,56,59]. One test finding in one study [55] found healthy controls to have significantly worse language than patients with glioma with medium effect (using Bedomning av subtila sprakstorningar Metaphors). Nineteen test findings across six studies [44–46,54,55,59,60] found no significant differences in language between patients and healthy controls (using Bedomning av subtila sprakstorningar, Picture-Sentence Matching Task, Spontaneous Speech, and Verbal Fluency Test). Fourteen non-significant findings [44,45,55,60] showed a small or medium effect. Six test findings in one study [54] reported no significance testing with five subtests of Picture-Sentence Matching Task showing higher scores in patients with glioma than controls, and one subtest showing similar scores.

Sub-group analysis of tumour location showed three studies [48,54,58] explored language in patients with only frontal or temporal lobe glioma, reporting nineteen test findings. Twelve of the nineteen test findings [48,54,58] demonstrated patients performed significantly worse than healthy controls. For the remaining seven test results [54], one showed that language did not differ between patients and controls, and six reported no significance testing but showed higher patient scores than control group scores.

#### Memory.

Seven studies [46,47,50,52,58,60,61] explored memory as a cognitive function, reporting twenty test results. Five test findings across three studies [52,60,61] found patients to have significantly worse memory than healthy controls, reporting medium and large effect sizes (using Auditory Verbal Learning Test, Memory with Interference (30s), Story Recall Immediate and Delayed, and Visual Verbal Learning Test). Effect size was not calculable for one finding [52] as patient and control scores were not reported. Fifteen test findings across five studies [46,47,50,58,60] found no significant differences in memory between patients and healthy controls (using Corsi, Memory Test, Munich Verbal Memory Test [Münchner Verbaler Gedächtnistest], Tapping Test, Visual Verbal Learning Test, and Wechsler Adult Intelligence Scale Working Memory). Ten non-significant findings [47,50,60] showed a small or medium effect. Three non-significant findings in two studies [50,58] showed a large negative effect, where patients performed worse than healthy controls.

Regarding tumour location, two studies [50,58] explored memory in patients with only frontal or temporal lobe glioma, reporting four test findings, none of which differed between patients and controls.

#### Information processing.

Two studies [51,59] explored information processing as a cognitive function, reporting sixteen test results. Four test findings across both studies [51,59] found patients to have significantly worse information processing than healthy controls, some with medium or large effect sizes (using Matching Tasks: Auditory, Picture Naming Task, and Sentence Judgement Test). Effect size was not calculable for one finding [59]. Nine test findings across two studies [51,59] found no significant differences in information processing between patients and healthy controls (using Learning-Meaningful and Non-Meaningful Tasks, Matching Tasks, and Sentence Judgement Test Time). Eight non-significant findings [51] showed a small or medium effect. Three test findings in one study [59] reported no significance testing and did not report scores for the control group (using Sentence-Judgement Test).

#### Executive function.

Five studies [46,50,52,57,61] explored executive function as a cognitive function, reporting eleven test results. Four test findings across four studies [50,52,57,61] found patients to have significantly worse executive function than healthy controls, some with medium or large effect (using Concept Shifting, Mini Mental State Examination, and Modified Card Sorting Test). Effect size was not calculable for two findings [52,57]. Seven test findings across two studies [46,50] found no significant differences in executive function between patients and healthy controls (using Concept Shifting, and subtests of Modified Card Sorting Test). Five non-significant findings [50] showed a small or medium effect, and one non-significant finding in the same study showed a large effect (using Modified Card Sorting Test: Preservative Error).

#### Decision-making.

One study [49] (which included patients with only frontal or temporal lobe glioma) explored decision-making in patients, reporting five test results. Four test findings found patients to have significantly worse decision-making than healthy controls, and had large effect sizes (using subtests of Gambling Task). In the remaining test, there was no significant difference in decision-making between patients and healthy controls showing a small effect (using subtest of Gambling Task).

#### Attention.

One study [60] explored attention as a cognitive function, reporting four test results. Two test findings found patients to have significantly worse attention than healthy controls, and two test findings found no significant differences in attention between patients and healthy controls (using subtests of Conner’s Continuous Performance Test). Effect size was not calculable.

#### Visuospatial function.

One study [58] (which included patients with only frontal or temporal lobe glioma) explored visuospatial function as a cognitive function, reporting two test results; both found patients to have significantly worse visuospatial function than healthy controls with large effect sizes (using Mapping, and Visuospatial tests).

#### Studies using the same cognitive function test.

The DST Total was used by two studies [50,58], and DST Forwards and Backwards each by two studies [50,60] and reported mixed results. The Boston Naming Test (BNT) was used by two studies [55,59]; both found patients with glioma performed significantly worse than healthy controls. The TMT A was used by three studies [59–61], and reported mixed results and selective outcome reporting; the TMT B was used by two studies [59,60] with mixed results and selective outcome reporting. Furthermore, Stroop tests were administered in four studies [46,52,60,61]; however, it was not clear which version was used across the four studies. Outcomes were not fully reported across all studies, resulting in an inability to combine findings across multiple studies. As a result of selective outcome reporting and heterogeneity in the methods used in the studies, a meta-analysis was not deemed appropriate.

## Discussion

This review assessed the literature exploring cognitive function in patients with glioma compared to healthy controls. Over the 17 studies analysed, the number of cognitive function tests (n = 143) and their findings, and the testing procedure and approach, were highly varied. This is a common finding in other literature exploring cognitive function in patients with glioma [62]. This heterogeneity made it challenging to draw firm conclusions. However, our review suggests that tests measuring multiple cognitive functions, and language in particular, have some potential to identify differences in the cognitive performance of patients with glioma and healthy controls, while acknowledging that cognitive function deficits are also highly prevalent in many neurological diseases.

The MoCA, Mapping, Dual-Attention Task Visual, Arithmetic, and DSST tests showed the largest effect sizes between patients with glioma and healthy controls. The Mapping and Arithmetic tests were not described in detail; therefore, it is not possible to determine accurately which specific measures were administered during the study [58]. The Dual-Attention Task Visual and DSST were administered by neuropsychologists. The Dual-Attention Task Visual was carried out on computer and preceded by a training phase. Individual tests, such as the DSST, generally require <5 minutes duration. No further specific information on time duration, setting, or administration was reported to assess the acceptability and feasibility of these tests for primary care application. These tests would need further investigation into required time, cost and administration resources. The BNT was administered across two studies [55,57] with large numbers of participants, showing medium to large effect size, suggesting this test has potential to identify cognitive deficits associated with glioma; however, within the context of this review, the BNT is likely to be too long (30–45 minutes) to administer within primary care [63].

The MoCA, which assesses multiple cognitive domains, is possibly one of the most suitable tests for primary care, with short test duration, and existing clinical application within primary care for other neurological conditions, such as dementia [64]. It also has the most promising findings in this review with the largest effect size of all included measures. Furthermore, the established cut-off score for the MoCA is < 26, and the patients in the included study scored a mean of 20.2 compared to a mean of 27.9 for the control group. While the established cut-off score appeared suitable for this sample, it is important to acknowledge the risk of false positives and potentially varying scores for other samples. The MoCA is mentioned as a commonly used clinical test in European Association of Neuro-Oncology (EANO) guidelines for diagnosis of glioma [65]. A recent systematic review [62] questioned the sensitivity of the MoCA for patients with glioma as it was not developed to detect subtle cognitive changes associated with brain tumours. Our current findings suggest that the MoCA might have potential to be capable of identifying cognitive differences in patients with glioma. The MoCA also has low time and cost demands, and high patient readability, consent, completion rates, and acceptability, and needs no specialist administration [62], suggesting it is a highly acceptable and feasible test for primary care. However, in this review, it must be acknowledged that the MoCA was used in a single study with a small sample size; this lack of a large body of evidence supporting the MoCA used in this context highlights the need for caution.

Certain cognitive function tests might be capable of discriminating between patients with glioma and healthy controls. Cognitive tests could provide support for GP decision-making around which patients would benefit from onward referral and investigation, either via an urgent suspected cancer pathway or a GP direct access to neuroimaging pathway. However, further exploration is required around feasibility and implementation of cognitive testing in the current brain tumour diagnostic pathway in primary care. Similarly, it is important to consider how these tests are administered and the clinical implications within primary care. For instance, the length of testing, mode of delivery, administration, eligibility, and required resources and workload [62]. Lengthy tests and specialised administrators are not feasible in primary care settings [66], given impact on resources and workload [67]. Practical challenges of time constraints, GP training, and clinical workflow considerations must be further explored. Tests that showed discriminatory utility, such as the Mapping, Dual-Attention Task Visual, Arithmetic, and DSST, need further investigation into time and cost resources and administration requirements for application in primary care. The MoCA, as an established and validated test used within primary care for other conditions, is potentially suitable for future implementation research to determine how the administration in primary care would impact on resources, workload, and outcomes from healthcare professional, patient and public perspectives [67]. External validation of cognitive tests would also be required, particularly in a primary care setting with relevant patient populations.

It is important to acknowledge that there is a disproportionate representation of patients with left hemisphere gliomas in the included studies. However, the MoCA, DSST, and Dual-Attention Task Visual were all tested in a mixed group of patients with equal representation of left- and right-hemisphere gliomas. Similarly, it might be presumed that cognitive function tests would be more likely to detect cognitive deficits in patients with high-grade glioma, as a more intrusive tumour, compared to low-grade glioma. However, the MoCA [53] was administered only to patients with low-grade glioma, and the Dual-Attention Task Visual [51] was tested equally in patients with low- and high-grade glioma. The grade of the tumour was not reported for patients who performed the DSST [58].

The results of this systematic review suggest cognitive function tests measuring multiple cognitive functions and language could have clinical utility for identifying a difference in cognitive function between patients with glioma and healthy control groups. This suggests language impairment is objectively detectable in patients with glioma, in line with previous research [7]. Indeed, there has been recent interest in language testing to support detection of glioma [66]. Verbal fluency testing was identified as predominantly measuring language function by most of the included studies in this review [44,45,48,55,56,60,61], though it could be argued that it measures other cognitive functions in conjunction with language function [66]. However, it is important to acknowledge the disproportionate number of cognitive function tests measuring language in the review, and the findings should be interpreted within this context. Tumour location might further influence multiple cognitive function or language impairment, given the high ratio of tests reporting significant differences in these domains for patients with frontal or temporal lobe tumours compared to controls. However, these findings should be interpreted with caution due to small sample sizes.

Memory deficits are often cited as a cognitive symptom associated with glioma [20,21]. However, most cognitive function tests in this review found no difference in memory performance between patients with glioma and healthy participants. These findings indicate memory function tests might not have clinical utility within this context. It also could be argued that different forms of memory should be studied, such as working memory and long-term memory. For other cognitive functions such as information processing, executive function, decision making, attention, and visuospatial function, the results were mixed or few in number. This limits conclusions about their utility. Cognitive function tests measuring executive function might be clinically useful; however, the mixed findings might reflect a lack of established consensus for defining executive function, which can be used as an umbrella term that includes attention, memory, and cognitive flexibility [40]. The definition of executive function as accepted in this review is that of general neurological function that is separate from more specialised cognitive functions, such as memory or cognitive flexibility.

Some non-significant findings showed a large effect whereby patients scored worse on the cognitive function tests than the healthy control group. In particular, the DST (measuring multiple cognitive functions), Tapping Test (measuring memory), and Modified Card Sorting Test (measuring executive function). These non-significant findings might be due to the small sample sizes and demonstrate the need for further robust exploration to draw any further conclusions about their utility.

Overall, the same cognitive function test was rarely used across multiple studies making comparisons difficult and limiting options to perform a meta-analysis. Consistency in choice of cognitive function test with patients with glioma is crucial to understand whether these tests have the capability of detecting differences in cognitive function. Replication studies are required to further this evidence base. The most promising areas are impairments in multiple cognitive function or language, and these could be the initial focus.

The strengths of this review include systematic searching of three databases, and the use of quality assessment methods. Searching these three databases is recommended by Cochrane [68] as providing comprehensive coverage of the relevant literature, though further databases could have been included to further the potential to identify eligible studies. Furthermore, no meta-analysis was carried out, so there is a strong need for caution when interpreting any conclusions. The results of the review are limited in that all control groups were healthy participants. In contrast, patients presenting to primary care are likely to be symptomatic, which might affect the applicability of these findings to primary care. Research is therefore needed to compare discrimination of cognitive function results in a population presenting to primary care with symptoms potentially associated with glioma, such as headache or weakness. Furthermore, this review did not include studies that compared data of patients with glioma to normative data, and only included case-control studies; this may have resulted in missing some studies that could contribute relevant findings. In addition, there is no clear agreement on the definition of different types of cognitive function. For instance, there is ongoing debate whether emotion recognition is included as a cognitive function. Studies testing emotion recognition were not included in this review but could offer additional insight into identification of cognitive differences between patients with glioma and healthy controls [69]. Furthermore, the secondary aim of this review to determine any adverse effects experienced by participants was not fulfilled as no data was provided by the included studies on this outcome.

Limitations of the included papers further limit the conclusions of this systematic review. The wide heterogeneity of the included studies, with regards methodology, sample sizes and demographics, and types of cognitive tests used, makes direct comparisons of findings challenging. For example, different cognitive tests might assess different cognitive domains and have varying levels of accuracy. Different patient samples could have varying socio-demographics as well as varying tumour histology and location, which could influence comparison across study findings. Furthermore, study design could vary in patient and control recruitment methods and cognitive test administration procedures. The heterogenous factors have been reported transparently in this review as this is likely to impact on the reliability of these conclusions [70]. Most studies were single-centre, with small sample sizes, potentially reducing representativeness and statistical power of the findings. Often, cognitive function tests were administered as part of standard clinical care, making it more difficult to extrapolate to uniform delivery within populations of patients with glioma, and between patients and controls. As such, it was also not always reported which specific test or which version of a test was being used. Furthermore, some studies did not report significance testing, which makes it difficult to determine the impact on the findings of this review. Some studies assessing memory, language, information processing, and executive function were lacking this data, though the reasons for this are unknown and require further investigation. Similarly, effect sizes were not calculable for all findings due to the lack of reported data. Over half the studies excluded patients with severe cognitive deficits, possibly resulting in underestimating differences in cognitive function. The selection and matching of control groups are not consistently reported. Without proper matching for age, education, and other factors, observed cognitive differences may be due to confounding rather than the presence of glioma. Furthermore, no studies reported ethnicity or socioeconomic status of participants, and as such, it is challenging to understand the representativeness of the samples, potentially reducing the generalisability of the findings. Future research should be widely applicable, using representative samples, specifically including patients with poorer prognoses, including those ineligible for surgical intervention, and with severe cognitive deficits, who are largely underrepresented in these samples. Similarly, further exploration, validation and diagnostic accuracy studies would be needed in a representative sample of the population with brain tumours, or indeed in a population of patients presenting to primary care with symptoms that might prompt a referral for neuroimaging. Future validation studies of cognitive function testing in primary care settings are also needed. Robust research would benefit from reporting full demographic data, including ethnicity and socioeconomic status for both patients and controls to improve representativeness and investigate health inequalities.

## Conclusions

In conclusion, this systematic review has emphasised the high variability with which cognitive function is assessed in studies with patients with glioma. Cognitive function tests might have utility to be used alongside guidelines for referral [71] to raise suspicion of glioma by identifying early change in cognitive function, in particular, within an overall multiple cognitive function domain or language function. However, high heterogeneity and the presence of risk of bias in existing studies means any interpretations and the reliability of the conclusions should be treated with caution. The applicability to primary care requires further exploration, in particular testing feasibility and acceptability of the cognitive function tests within the workflow and capacity of primary care.

## Supporting information

S1 Table Search strategy. (DOCX)

S2 File Cognitive function measured by each cognitive function test: process. (DOCX)

S3 Table Numbered table of eligible studies and data extraction process. (DOCX)

S4 Table Supplementary summary of study aims and participant characteristics in systematic review. (DOCX)

S5 Table Supplementary summary of participant characteristics and study administration. (DOCX)

S6 Table Summary of studies that include patients with frontal or temporal location of glioma in systematic review. (DOCX)

S7 Table Matrix showing which cognitive function was tested in which studies with only frontal or temporal lobe glioma and whether a significant difference was found between patients and control groups. (DOCX)

S8 Table PRISMA 2020 checklist. (DOCX)

S9 File Protocol. (PDF)

S10 Table Full text review. (XLSX)
