# Supplementary material for: Can cognitive function tests discriminate between patients with glioma and healthy controls prior to treatment? A systematic review
Source: PLoS One. 2025 Aug 6;20(8):e0329663. doi: 10.1371/journal.pone.0329663 (PMC12327679; doi:10.1371/journal.pone.0329663)
Supplement: S1 Table — (DOCX) [file pone.0329663.s001.docx]

S1 Table. Search strategy

Most recent search: 3^rd^ July 2024

| **Cognitive function tests that discriminate between patients with and without brain tumour** | | |
| --- | --- | --- |
| Numbers 1 and 2 are the keywords and MeSH terms in the title or abstract for the **Population** and are combined using **OR** | 1. Brain tumour* or brain cancer* 2. "Brain Neoplasms"[Mesh] | Population (P) |
| Numbers 3 and 4 are the keywords and MeSH terms in the title or abstract for the **Intervention** and are combined using **OR** | 1. Cognit* function* test* OR cognit* function* assessment* OR cognitive function* exam* OR executive function* test* OR executive function* assessment* OR executive function* exam* OR neuropsycholog* assessment* OR neuro-psycholog* assessment* OR neuropsycholog* exam* OR neuro-psycholog* exam* OR cognit* test* OR cognit* assessment* OR cognit* exam* OR cognit* abilit* test* OR clock-drawing test OR Montreal cognitive test OR mini-mental state exam OR abbreviated mental test OR memory impairment screen OR mental status questionnaire OR short portable mental status questionnaire OR neuropsychiatric inventory questionnaire OR mini examen cognoscitivo OR Eurotest OR Fototest OR memory alteration test   OR verbal fluency OR memory OR mental capacity   1. "Neuropsychological Tests"[Mesh] | Intervention (I) |
| **P** and **I** keywords and MeSH terms combined using **AND** |  | P and I combined |

**Pilot Search**

**MEDLINE (via PubMed)**

((Brain tumour*[Title/Abstract]) OR (brain cancer*[Title/Abstract]) OR (brain neoplasms[MeSH Terms])) AND ((Cognit* function* test*[Title/Abstract]) OR (cognit* function* assessment*[Title/Abstract]) OR (cognitive function* exam*[Title/Abstract]) OR (executive function* test*[Title/Abstract]) OR (executive function* assessment*[Title/Abstract]) OR (executive function* exam*[Title/Abstract]) OR (neuropsycholog* assessment*[Title/Abstract]) OR (neuro-psycholog* assessment*[Title/Abstract]) OR (neuropsycholog* exam*[Title/Abstract]) OR (neuro-psycholog* exam*[Title/Abstract]) OR (cognit* test*[Title/Abstract]) OR (cognit* assessment*[Title/Abstract]) OR (cognit* exam*[Title/Abstract]) OR (cognit* abilit* test*[Title/Abstract]) OR (clock-drawing test[Title/Abstract]) OR (Montreal cognitive test[Title/Abstract]) OR (mini-mental state exam[Title/Abstract]) OR (abbreviated mental test[Title/Abstract]) OR (memory impairment screen[Title/Abstract]) OR (mental status questionnaire[Title/Abstract]) OR (short portable mental status questionnaire[Title/Abstract]) OR (neuropsychiatric inventory questionnaire[Title/Abstract]) OR (mini examen cognoscitivo[Title/Abstract]) OR (Eurotest[Title/Abstract]) OR (Fototest[Title/Abstract]) OR (memory alteration test[Title/Abstract]) OR (verbal fluency[Title/Abstract]) OR (memory[Title/Abstract]) OR (mental capacity[Title/Abstract]) OR (neuropsychological test[MeSH Terms]))

**CENTRAL**

(Mesh brain neoplasms) OR brain tumour* or brain cancer* in Title Abstract Keyword AND (Mesh neuropsychological tests) OR cognit* function* test* OR cognit* function* assessment* OR cognitive function* exam* OR executive function* test* OR executive function* assessment* OR executive function* exam* OR neuropsycholog* assessment* OR neuro-psycholog* assessment* OR neuropsycholog* exam* OR neuro-psycholog* exam* OR cognit* test* OR cognit* assessment* OR cognit* exam* OR cognit* abilit* test* OR clock-drawing test OR Montreal cognitive test OR mini-mental state exam OR abbreviated mental test OR memory impairment screen OR mental status questionnaire OR short portable mental status questionnaire OR neuropsychiatric inventory questionnaire OR mini examen cognoscitivo OR Eurotest OR Fototest OR memory alteration test OR verbal fluency OR memory OR mental capacity in Title Abstract Keyword - (Word variations have been searched)

**EMBASE (with all synonyms included)**

('brain tumor'/exp OR 'brain neoplasm' OR 'brain neoplasms' OR 'brain supratentorial tumor' OR 'brain supratentorial tumour' OR 'brain tumor' OR 'brain tumor diagnosis' OR 'brain tumour' OR 'brain tumour diagnosis' OR 'cerebral tumor' OR 'cerebral tumour' OR 'cerebroma' OR 'cerebrum tumor' OR 'cerebrum tumour' OR 'encephalophyma' OR 'intracerebral tumor' OR 'intracerebral tumour' OR 'intracranial neoplasm' OR 'midline tumor' OR 'midline tumour' OR 'multiple brain tumor' OR 'multiple brain tumour' OR 'subtentorial tumor' OR 'subtentorial tumour' OR 'supratentorial brain tumor' OR 'supratentorial brain tumour' OR 'supratentorial neoplasms' OR 'supratentorial tumor' OR 'supratentorial tumour' OR 'tumor cerebri' OR 'tumor, brain' OR 'tumour cerebri' OR 'tumour, brain' OR 'brain cancer'/exp OR 'brain cancer' OR 'brain carcinoma' OR 'brain malignant tumor' OR 'brain malignant tumour' OR 'carcinoma, brain' OR 'carcinoma, cerebral' OR 'cerebral carcinoma' OR 'cerebral neoplasm') AND ('cognitive function test'/exp OR 'cognition test' OR 'cognitive abilities test' OR 'cognitive ability test' OR 'cognitive function test' OR 'cognitive functioning test' OR 'cognition assessment'/exp OR 'cognition assessment' OR 'cognitive function assessment' OR 'memory and learning tests' OR 'executive function test'/exp OR 'executive function task' OR 'executive function test' OR 'problem solving task' OR 'problem solving test' OR 'neuropsychological test'/exp OR 'neuro-psychological assessment' OR 'neuro-psychological examination' OR 'neuro-psychological test' OR 'neuropsychologic test' OR 'neuropsychological assessment' OR 'neuropsychological examination' OR 'neuropsychological test' OR 'neuropsychological tests' OR 'neuropsychology test' OR 'test, neuropsychological' OR 'cognitive assessment'/exp OR 'clock drawing test'/exp OR 'montreal cognitive assessment'/exp OR 'montreal cognitive assessment' OR 'mini mental state examination'/exp OR 'mmse' OR 'mini mental state examination' OR 'mini-mental state examination' OR 'abbreviated mental test'/exp OR 'memory impairment screen'/exp OR 'short portable mental status questionnaire'/exp OR 'spmsq' OR 'short portable mental status questionnaire' OR 'short portable mental status examination' OR 'short portable mental status score' OR 'neuropsychiatric inventory questionnaire'/exp OR 'memory alteration test'/exp OR 'verbal fluency'/exp OR 'memory'/exp OR 'item recall' OR 'memory' OR 'memory function' OR 'nonspatial memory' OR 'remembering' OR 'reminiscence' OR 'mental capacity'/exp OR 'ability, mental' OR 'attainment' OR 'capacity, mental' OR 'fitness, mental' OR 'mental ability' OR 'mental capacity' OR 'mental competency' OR 'mental fitness')
