# Supplementary material for: Can cognitive function tests discriminate between patients with glioma and healthy controls prior to treatment? A systematic review
Source: PLoS One. 2025 Aug 6;20(8):e0329663. doi: 10.1371/journal.pone.0329663 (PMC12327679; doi:10.1371/journal.pone.0329663)
Supplement: S2 File — (DOCX) [file pone.0329663.s002.docx]

**S2. Cognitive Function Measured by Each Cognitive Function Test: Process**

All cognitive function tests used across all included studies were listed alongside the cognitive function(s) each test was measuring, as reported by each study. This was compared across all included studies to ensure consistency. Existing literature using the same tests was consulted where there was uncertainty or conflicts. Where there was conflict in the claimed measured cognitive function, the majority consensus was adopted and validated using existing literature. This resulted in review-determined cognitive functions measured for each cognitive function test (see Table 6). Each cognitive function test was categorised as measuring multiple cognitive functions or one of following cognitive functions:

- Language: Language refers to semantic and phonemic processing and comprehension[1], and includes definitions, sentence analysis, verbal naming, repetition, inference, semantic selection, story and sentence completion, and letter and category fluency.
- Memory: Memory is a complex cognitive function that encompasses subsets of memory function[2], including short-term and long-term, verbal and visual, and working memory.
- Information processing: Information processing is defined as the processing of sensory and perceptual information in order to evoke a response based on world knowledge[3], and includes processing speed and perceptual reasoning.
- Executive function: Executive function is often used as a term to encompass multiple different cognitive functions[4], including distinct cognitive functions of memory[5] and cognitive flexibility[6]; however, some cognitive function tests in the included studies were claimed to measure executive function distinctly. The definition of executive function as accepted in this review is that of general neurological function that is separate from more specialised cognitive functions[7]. Where validated, cognitive functions that are sometimes included under the general term of executive function were used as standalone cognitive functions, in particular, memory and cognitive flexibility.
- Decision making: Decision-making is considered to be making decisions based on potential consequences and benefits in the immediate and / or long-term[8].
- Attention: Attention is defined as the cognitive ability to represent and maintain contextual information that is required to inform appropriate decisions or behaviours[9], and includes concentration, verbal and auditory, and sustained attention.
- Visuospatial function: Visuospatial function is defined as the application of visuo-constructive abilities, spatial awareness alongside perception, and conceptualisation[10].

S7 Table. Review-determined cognitive functions measured by each cognitive function test used across the included studies in this systematic review.

| **Study** | **Cognitive Test(s)** | **Cognitive Function (as reported by study)** | **Review Determined Cognitive Function** |
| --- | --- | --- | --- |
| Reijneveld et al 2001[46] | Visual Verbal Learning test  Working memory task | Memory  Attention  Executive function  Psychomotor functioning | Memory |
|  | Categoric word fluency |  | Language |
|  | Letter-Digit Substitution test |  | Multiple cognitive functions[5] |
|  | Stroop Color-Word test |  | Multiple cognitive functions[11] |
|  | Concept shifting test |  | Executive function[12] |
| Ruge et al 2010[47] | Munchner Verbaler Gedachtnistest (MVGT) | Memory | Memory |
| Bizzi et al 2012[48] | Aachenie Aphasie (AAT) Token test  AAT Written language  AAT Comprehension  AAT Communicative behaviour  Semantic Fluency  Phonemic Fluency  Spontaneous Speech | Language | Language |
| Mattavelli et al 2012[49] | Gambling task | Decision-making | Decision-making |
| Mu et al 2012[50] | Digit Span Total  Digit Span Test backward  Tapping Test | Memory | Multiple cognitive functions[13] |
|  | Digit Span Test forward | Attention | Memory[14] |
|  | Modified Card Sorting Test | Executive function | Executive function |
| Plaza et al 2013[51] | Matching Tasks  Learning–Meaningful Tasks  Learning–Nonmeaningful Tasks | Information processing | Information processing |
|  | Dual–Attention Task | Attention and memory | Multiple cognitive functions |
|  | Picture–Naming Task | Processing speed | Processing speed |
| Satoer et al 2013[44] *and* 2018[45] | Spontaneous Speech  Boston Naming Test  Category Fluency | Language | Language |
| Habets et al 2014[52] | Verbal Learning Test | Memory | Memory |
|  | Concept Shifting Test (condition A, B, C, categoric word fluency test) | Executive function | Executive function |
|  | Concept Shifting Test (condition 0) | Psychomotor function | Executive function[12] |
|  | Letter Digit Modalities Test | Information processing speed | Multiple cognitive functions[5] |
|  | Stroop Word-Color Test | Attention | Multiple cognitive functions[11] |
| Huang et al 2014[53] | MoCA | n.d. | Multiple cognitive functions[15] |
| Kinno et al 2014[54] | Picture-sentence matching task | Language | Language |
| Antonsson et al 2018[55] | BeSS Total  BeSS Repetition of Long Sentences  BeSS Recreating Sentences  BeSS Making inferences  BeSS Comprehension of logico-grammatical sentences BeSS comprehension of ambiguous sentences  BeSS comprehension of metaphors  BeSS Word definitions  Sentence analysis  Morphological completion  Boston Naming Test  FAS  Animals – Word Fluency  Verbs – Word Fluency | Language | Language |
| De Witte et al 2018[56] | TeleLanguage Test A and B | Language | Language |
| Zhang et al 2018[57] | BNT  AQ  Aphasia Battery for Chinese Speakers (ABC) | Language | Language |
|  | MMSE |  | Executive function[4] |
| Hu et al 2020[58] | Visuospatial Test  Mapping | n.d. | Visuospatial function |
|  | Math Exam |  | Multiple cognitive functions[7] |
|  | Similarity |  | Language[16] |
|  | DST  Memory Test |  | Memory[14] |
|  | DSST |  | Multiple cognitive functions[5] |
| Mooijman et al 2022[59] | Sentence Judgement Test | Processing speed | Processing speed |
|  | Boston Naming test  Token test | Language | Language |
|  | TMT A | Visuo-perceptual speed | Multiple cognitive functions[6] |
|  | TMT B | Concept-shifting |  |
|  | TMT BA | Cognitive flexibility |  |
| Tarantino et al 2022[60] | Digit Span forward  Digit Span backward | Memory and attention | Multiple cognitive functions[14] |
|  | Immediate story recall  Delayed story recall  Memory with interference  Corsi block-tapping forward  Corsi block-tapping backward | Memory | Memory |
|  | Boston Naming test  Phonemic fluency | Language | Language |
|  | TMT A  TMT B | Processing speed and executive function | Multiple cognitive functions |
|  | Stroop Color test |  | Multiple cognitive functions[11] |
|  | AX-CPT | Proactive and reactive cognitive control | Attention [9] |
| Wang et al 2022[61] | Trail Making Test A (TMT A) | Processing speed | Multiple cognitive functions[6] |
|  | Stroop Test B |  | Multiple cognitive functions[11] |
|  | Auditory Verbal Learning Test (AVLT) | Memory | Memory |
|  | Mini Mental State Examination (MMSE) (Chinese version) | Executive function | Executive function |
|  | Stroop Test C–B |  | Multiple cognitive functions[11] |
|  | Trail Making Test B–A (TMT BA) |  | Multiple cognitive functions[6] |
|  | Verbal Fluency Test (VFT)  Boston Naming Test (BNT) | Language | Language |
|  | Symbol Digit Modalities Test (SDMT) | Attention | Multiple cognitive functions[5] |

(Author paper references(44–61) refer to references in main report)

**References**

1. Weng SM, Fang SY, Li LW, Fan X, Wang YY, Jiang T. Intra-operative mapping and language protection in glioma. Chin Méd J. 2021;134(20):2398–402.

2. Durand T, Berzero G, Bompaire F, Hoffmann S, Léger I, Jego V, et al. Episodic Memory Impairments in Primary Brain Tumor Patients. Arch Clin Neuropsychol [Internet]. 2018 Dec 1;33(8):949–55. Available from: <http://dx.doi.org/10.1093/arclin/acx138>

3. Plaza M, Capelle L, Maigret G, Chaby L. Strengths and weaknesses of multimodal processing in a group of adults with gliomas. Neurocase. 2013;19(3):302–12.

4. Arevalo‐Rodriguez I, Smailagic N, Figuls MR i, Ciapponi A, Sanchez‐Perez E, Giannakou A, et al. Mini‐Mental State Examination (MMSE) for the detection of Alzheimer’s disease and other dementias in people with mild cognitive impairment (MCI). Cochrane Database Syst Rev. 2015;(3):CD010783.

5. Jaeger J. Digit Symbol Substitution Test. J Clin Psychopharmacol. 2018;38(5):513–9.

6. Bowie CR, Harvey PD. Administration and interpretation of the Trail Making Test. Nat Protoc. 2006;1(5):2277–81.

7. Menon V. Developmental cognitive neuroscience of arithmetic: implications for learning and education. ZDM. 2010;42(6):515–25.

8. Mattavelli G, Casarotti A, Forgiarini M, Riva M, Bello L, Papagno C. Decision-making abilities in patients with frontal low-grade glioma. J Neurooncol. 2012;110(1):59–67.

9. Smid HGOM, Witte MR de, Homminga I, Bosch RJ van den. Sustained and Transient Attention in the Continuous Performance Task. J Clin Exp Neuropsychol. 2006;28(6):859–83.

10. Morshed RA, Young JS, Kroliczek AA, Berger MS, Brang D, Hervey-Jumper SL. A Neurosurgeon’s Guide to Cognitive Dysfunction in Adult Glioma. Neurosurgery [Internet]. 2021 Jun 15;89(1):1–10. Available from: <http://dx.doi.org/10.1093/neuros/nyaa400>

11. Scarpina F, Tagini S. The Stroop Color and Word Test. Front Psychol. 2017;8:557.

12. Elst WV der, Boxtel MPJV, Breukelen GJPV, Jolles J. The Concept Shifting Test: Adult Normative Data. Psychol Assess. 2006;18(4):424–32.

13. Digit Span (DGS) - Cambridge Cognition [Internet]. [cited 2024 Jun 14]. Available from: <https://cambridgecognition.com/digit-span-dgs/>

14. Woods DL, Kishiyama MM, Yund EW, Herron TJ, Edwards B, Poliva O, et al. Improving digit span assessment of short-term verbal memory. J Clin Exp Neuropsychol. 2011;33(1):101–11.

15. Nasreddine ZS, Phillips NA, Bédirian V, Charbonneau S, Whitehead V, Collin I, et al. The Montreal Cognitive Assessment, MoCA: A Brief Screening Tool For Mild Cognitive Impairment. Journal of the American Geriatrics Society [Internet]. 2005;53(4):695–9. Available from: <http://onlinelibrary.wiley.com/doi/abs/10.1111/j.1532-5415.2005.53221.x>

16. Fernaeus SE, Hellström Å. Conceptual elaboration versus direct lexical access in WAIS-similarities: differential effects of white-matter lesions and gray matter volumes. Aging, Neuropsychol, Cogn. 2018;25(6):893–903.
