# Supplementary material for: Can cognitive function tests discriminate between patients with glioma and healthy controls prior to treatment? A systematic review
Source: PLoS One. 2025 Aug 6;20(8):e0329663. doi: 10.1371/journal.pone.0329663 (PMC12327679; doi:10.1371/journal.pone.0329663)
Supplement: S3 Table — (DOCX) [file pone.0329663.s003.docx]

Table S3: Numbered table of eligible studies and data extraction process

| **Study** | **Included in Analysis** | **Data Extraction** | | **Eligible** |
| --- | --- | --- | --- | --- |
|  |  | **Date** | **Name** |  |
| 1. Reijneveld et al 2001[46] | Yes | August 2023 | LS, SS, RC | Yes |
| 2. Ruge et al 2010[47] | Yes | August 2023 | LS, RC | Yes |
| 3. Bizzi et al 2012[48] | Yes | August 2023 | LS, SS | Yes |
| 4. Mattavelli et al 2012[49] | Yes | August 2023 | LS, RC | Yes |
| 5. Mu et al 2012[50] | Yes | August 2023 | LS, RC | Yes |
| 6. Plaza et al 2013[51] | Yes | October 2023 | LS, RE | Yes |
| 7. Satoer et al 2013[44] *and* 2018[45] | Yes | August 2023 | LS, RC | Yes |
| 8. Habets et al 2014[52] | Yes | August 2023 | LS, RC | Yes |
| 9. Huang et al 2014[53] | Yes | August 2023 | LS, RC | Yes |
| 10. Kinno et al 2014[54] | Yes | August 2023 | LS, RC | Yes |
| 11. Antonsson et al 2018[55] | Yes | October 2023 | LS, RE | Yes |
| 12. De Witte et al 2018[56] | Yes | August 2023 | LS, SS | Yes |
| 13. Zhang et al 2018[57] | Yes | August 2023 | LS, RC | Yes |
| 14. Hu et al 2020[58] | Yes | August 2023 | LS, RC | Yes |
| 15. Mooijman et al 2022[59] | Yes | August 2023 | LS, RC | Yes |
| 16. Tarantino et al 2022[60] | Yes | August 2023 | LS, RC | Yes |
| 17. Wang et al 2022[61] | Yes | August 2023 | LS, SS | Yes |
| 18. Baxendale et al 2013^37^ | No  (Excluded for control group with hippocampal sclerosis and high risk of bias) | August 2023 | LS, RC | Yes |

Authors: LS: Laura Standen; SS: Suzanne Scott, RC: Roisin Curran, RE: Ruth Evans

n.d. No data reported
