# Supplementary material for: Can cognitive function tests discriminate between patients with glioma and healthy controls prior to treatment? A systematic review
Source: PLoS One. 2025 Aug 6;20(8):e0329663. doi: 10.1371/journal.pone.0329663 (PMC12327679; doi:10.1371/journal.pone.0329663)
Supplement: S4 Table — (DOCX) [file pone.0329663.s004.docx]

S4 Table. Supplementary summary of study aims and participant characteristics in systematic review

| **Study** | **Study Aim** | **Patient Characteristics** | | | | | | **Control Characteristics** | | | | | |
| --- | --- | --- | --- | --- | --- | --- | --- | --- | --- | --- | --- | --- | --- |
|  |  | **Age**  **mean (SD) years^e^** | **Sex**  **F / M**  **%** | **Education**  **Years (SD)^e^** | **Inclusion Criteria** | **Exclusion Criteria** | **Recruitment** | **Age**  **mean (SD) years^e^** | **Sex**  **F / M**  **%** | **Education**  **Years (SD)^e^** | **Inclusion Criteria** | **Exclusion Criteria** | **Recruitment** |
| Reijneveld et al 2001[46] | To determine the cognitive performance status and quality of life of patients with suspected low-grade glioma and investigate whether there is a difference in cognitive performance status and quality of life between suspected and proven low-grade glioma patients | n.s.^a^ | n.s.^a^ | n.s.^a^ | - Non-enhancing supratentorial lesions on MRI or CT without edema or mass effect - 18+ years old | - Signs of progression for 6+ months from diagnosis - Corticosteroids - Neurologic deficits | 16 hospitals in the western and central part of the Netherlands | 42.8 (11.6) | 43% / 57% | Level 4.5 (Dutch scoring system) | n.s. | n.s. | Database of the University of Maarstricht and the Netherlands Organization for Applied Scientific Research |
| Ruge et al 2010[47] | To clarify the status of cognitive functioning and HRQL of adult patients with low-grade glioma; to explore potential correlations between cognitive functioning, HRQL, and disease-related / epidemiological parameters | 44.4 (11.2) | 52% / 48% | n.s. | n.s. | n.s. | Department of Neurosurgery at Ludwig-Maximilians University | n.s. | n.s. | n.s. | n.s. | n.s. | Data pool from neuro-psychology group at the Department of Physical Medecine and Rehabilitation at the Ludwig-Maximilians University |
| Bizzi et al 2012[48] | To characterise the anatomy of language deficits in patients with brain tumours | 48.84 | 37% / 63% | 12.21 | - Pathological diagnosis of cerebral glioma - Right-handed - Scheduled surgery within one week of functional and diffusion tensor MRI - Able to perform fMRI and neuropsychological tasks | - <18 years old - Non-compliance during fMRI study - Diagnosis other than glioma - No neurological disease, psychiatric disorder or substance abuse | Consecutive patients | 35.5 (8) | 40% / 60% | 15 (2.1) | Right-handed | n.s. | n.d. |
| Mattavelli et al 2012[49] | To investigate decision-making in patients with a frontal low-grade glioma by using a more difficult version that the original Iowa Gambling Task; to verify effects of individual personality differences | 42.14 (10.94) | 50% / 50% | 13.05 (3.29) | Fluent spontaneous speech, word repetition, comprehension, and naming | n.s. | Patients in the neurosurgery Ward of the IRCCS Ospedale Maggiore Policlinico Mangiagalli-Regina Elena, Milan | 35.81 (16.06) | 54% / 46% | 14.73 (2.97) | n.d. | Neurologically impaired | n.d. |
| Mu et al 2012[50] | To clarify the performance of patients with a left-sided frontal glioma with regards to working memory and facial expression identification; to provide a reference for future rehabilitation strategies to improve the HRQoL of patients with brain tumours | 37  range 22–57 | 36% / 64% | 10.81 | - Primary tumour confined to the left frontal lobe, confirmed by a senior neurosurgeon based on clinical symptoms and imaging data - Postoperative diagnosis of glioma confirmed by a senior neuro-pathologist - 22–60 years old | - Deficits of intelligence, vision, hearing, language comprehension and expression - Neurological comorbidities - History of nervous system - Psychiatric diseases - Brain trauma - Diseases of other systems determined by interviews, MRI, surgical reports, neurological examination, and neuropsychological assessment | Department of Neurosurgery/Neuro-oncology at the Sun Yat-sen University Cancer Center | 36.73 (11.2) | n.s. | 10.72 | n.s. | n.s. | n.d. |
| Plaza et al 2013[51] | To describe patients’ multimodal processing that would be spared, altered or impaired by gliomas that slowly infiltrate various and diversely localised areas in cerebral hemispheres after accounting for task specificity and individual performances of patients compared to controls; to examine the correlation between localisation and impairments; to identify which tasks were most sensitive to tumour infiltration and plasticity limits | 31.8 (3.9) | 50% / 50% | 16.3 (2.4) | n.s. | n.s. | Neurosurgery Department if the Pitie-Salpetriere Hospital, Paris France | 29.3 (4.1) | 50% / 50% | 17.2 (2.8) | n.s. | n.s. | Paris community members and patients' relatives |
| Satoer et al 2013[44] *and* 2018[45] | To examine spontaneous speech of glioma patients pre- and post-operatively with usual standardised language tasks and tumour-specific characteristics  *and*  To investigate spontaneous speech of patients until long-term after glioma surgery in eloquent areas, taking into account tumour-related variables | 41.52  range 19–74  *and*  37.86  range 19–62 | 33% / 67%  *and*  18% / 72% | mode 5  4–7  Verhage system | - Eloquent brain areas - MRI diagnosis with no contrast enhancement - 18–65 years old - Native (or fluent) Dutch | - History of a medical, neurological or psychiatric condition known to affect cognition - History of substance abuse - Permanent cognitive or motor problems | n.d. | 39.44  range 19–62 | 62% / 38% | mode 5  4–7  Verhage system | n.s. | History of neurological disease | n.d. |
| Habets et al 2014[52] | To examine tumour and resection effects on cognition in a group of high-grade glioma patients | 60.6 (12.5) | 39% / 61% | 3.7 (2.2)  Code system 1–8 | Adult patients planned to undergo total or subtotal tumour resection for a radiologically suspected HGG | - History of neurological or severe psychiatric disorder potentially interfering with cognitive functioning - Insufficient command of the Dutch language | Medical Centre Haaglanden | n.s. | n.s. | n.s. | n.s. | n.s. | n.d. |
| Huang et al 2014[53] | To investigate if brain tumour patients have small-world topological property before and after operation under resting-state | 43.2 (13.1) | 58% / 42% | Primary n=4  Secondary n=5  High school n=2  Graduate n=1 | - Frontal lobe low-grade glioma (6x5x3.5cm) - Tumour extension not reaching central sulcus - Little edema in peritumour area - Slight enhancement of tumour in CT or MRI with contrast | Brain injury | Nanjing Brain Hospital | 41.8 (12.9) | 50% / 50% | Primary n=3  Secondary n=5  High school n=3  Graduate n=1 | n.s. | n.s. | Hospital staff at Nanjing Brain Hospital |
| Kinno et al 2014[54] | To analyse activation patterns of agrammatic patients with a left frontal glioma to identify any cortical reorganization of syntax-related networks | 34.33 (14) | 43% / 57% | n.s. | - Left frontal glioma - Right-handed - Completion of 3+ fMRI without significant head movement - Native Japanese | - Deficits in verbal / written communication or other cognitive abilities - Neurological or psychiatric disorders - Seizures - Medical problems related to MRI | University of Tokyo, Komaba | n.s. | n.s. | n.s. | n.s. | n.s. | n.d. |
| Antonsson et al 2018[55] | To explore language ability of patients with low-grade glioma using a language test battery; whether they reported any changes in their language, speech, or communicative ability before receiving treatment; whether the occurrence of language impairment differed depending on location | median 44  IQR 19 | 35% / 65% | median 15  IQR 5 | - Presumed low-grade glioma | - Moderate or severe developmental language or cognitive disorders - Non-native Swedish - Previous brain surgery and / or other tumour treatments - High-grade glioma | Patients with presumed LGG who presented at neurosurgical department of Sahlgrenska University hospital in Gothenburg | median 46  IQR 23 | n.s. | median 15  IQR 4 | n.s. | Neurological disease | Control group taken from larger study group |
| De Witte et al 2018[56] | To compose a feasible telephone-based language test battery for brain tumour patients; to collect longer follow-up data; to validate the telephone-based language battery with traditional face-to-face assessments | 55.14  range 39–68 | 29% / 71% | 15.5  range 12–19 | Left hemisphere with intraoperative language mapping during resection | - Comprehension disorders with inability to perform tests via telephone - Non-fluent English - History of medical or psychiatric condition affecting language - Language deficits due to prior treatment (<4 for comprehension screening test) - Auditory or severe visual disorder - Developmental delay | Patients  Hospital of California San Francisco Medical Center | 55.29 (10.33) | 29% / 71% | 15.64 (2.24) | Matched for age, gender, and education level | - Psychiatric diseases - Alcohol and / or drug addiction - Sleep medication - Impaired hearing or vision - Non-fluent English speakers | n.d. |
| Zhang et al 2018[57] | To localise the cerebellar regions with altered ALFF to identify the alteration of cerebellum neural activity; the correlation between structural and functional alteration; the altered pattern of cerebro-cerebellar circuit; to explore correlations of linguistic / cognitive scores and neuroimaging metrics for clinical significance | 39.73 (12.58) | 42% / 58% | 12.36 (2.96) | - Pathological glioma in left cerebral hemisphere - Location overlaps or within language network areas - 18–75 years old - Right-handed - Chinese Han nationality - 9+ years education - Imaging covered whole brain - Cooperated in cognitive evaluation | - Major neurological or psychiatric disorders - Alcohol or drug abuse - Motor impairment - No brain operation - Midline shift in structural images | Patient admitted in the Glioma Surgery Division of Huashan Hospital's Neurosurgery Department | 34.57 (13.11) | 48% / 52% | 12.98 (3.51) | - 18–75 years old - Right-handed - Chinese Han nationality - 9+ years of education - Cooperated in cognitive evaluation | - Motor impairment - Brain operation - Major neurological or psychiatric disorders   Alcohol or drug abuse | Advert |
| Hu et al 2020[58] | To investigate the alteration in contralateral GM volume, and the relationship between the alterations in contralateral GM volume with cognitive function in glioma patients | n.s.^b^ | n.s.^c^ | n.s.^d^ | - Histopathological unilateral temporal glioma - Native Chinese descent | - History of drug, alcohol abuse or substance abuse - History of head injury - Contraindications for participating in an MRI study - Neuropsychiatric illness and no illicit drug use over the past month | Department of Neurosurgery in Affiliated Brain Hospital of Nanjing Medical University, Jiangsu province, China | 56.82 (7.74) | 64% / 36% | 11.29 (3.53) | - Native Chinese descent | - No brain disorders or diseases - History of severe systemic disease, head trauma or psychological disorder | n.d. |
| Mooijman et al 2022[59] | To investigate whether response speed is a sensitive measure for self-reported word-finding problems in patients with a glioma; whether self-reported word-finding problems can be explained by lexical retrieval, linguistic processing speed or non-verbal cognitive abilities; whether linguistic processing speed is related to non-linguistic cognitive abilities | 45.37  range 18–73 | 33% / 67% | 5.36  range 3–7  Verhage system | - Awake surgery - Native Dutch | n.s. | Erasmus MC University Medical Centre | 42.75  range 19–61 | 57% / 43% | 5.53  range 3–7  Verhage system | - Normal vision and hearing | - History of cardiovascular, neurological, psychiatric or developmental language disorders - Substance abuse - Sleep medication, psychotropic or neuroleptic drugs | n.d. |
| Tarantino et al 2022[60] | To characterise cognitive control functions in patients with brain tumours; to explore the relationship between brain areas and the use of control mechanisms | 55.9 (13.6) | 36% / 64% | 11.6 (3.5) | - Clinical diagnosis of glioma - 18–85 years old - Preserved verbal comprehension, hand dexterity, visual acuity, and colour vision | - Metastases - Meningioma - Tumour recurrence - History of neurological or psychiatric disorders - Prior brain surgery - Medical conditions precluding active participation in research / altering interpretation of data - Inability to maintain wakefulness during tests - Insufficient Italian | Candidates for surgery  Neurology and Neurosurgery Clinics of the University Hospital of Padua | 55.2 (11.1) | 65% / 35% | 11.9  (3.4) | n.s. | - Neurological / psychiatric disorders   Difficulties in verbal comprehension, hand dexterity, visual acuity, or colour vision | n.d. |
| Wang et al 2022[61] | To assess cognitive function in patients with supratentorial glioma; factors affecting cognitive impairment to detect changes during treatment | 43.4 | 46% / 54% | 11.9 | - Supratentorial gliomas diagnosed by MRI - Pathological glioma - 18+ age - Right-handed - American Society of Anesthesiologists grade I-III - Completion of cognitive testing | - History of mental illness, alcohol abuse, previous stroke, hemiparesis and aphasia   Recurrent glioma | Patient presenting at hospital | 42.8 | 46% / 54% | 12.9 | n.s. | n.s. | Volunteer at the hospital |

^a^ Demographics reported for full sample only

^b^ Left: 57.25 (7.52) range 47–70; Right: 51.56 (17.56) range 21–69

^c^ Left: 25% / 75%; right: 33% / 67%

^d^ Left: 7.13 (3.91) range 0–12; right: 8.33 (3.91) range 0–12

**^e^** Range included where SD not reported

n.s. Not specified

(References refer to references in main report)
