# Supplementary material for: Can cognitive function tests discriminate between patients with glioma and healthy controls prior to treatment? A systematic review
Source: PLoS One. 2025 Aug 6;20(8):e0329663. doi: 10.1371/journal.pone.0329663 (PMC12327679; doi:10.1371/journal.pone.0329663)
Supplement: S5 Table — (DOCX) [file pone.0329663.s005.docx]

S5 Table. Supplementary summary of participant characteristics and study administration

| **Study** | **Comorbidities** | | **Other Relevant Tests** | | **Reasons for Missing Data** |
| --- | --- | --- | --- | --- | --- |
|  | **Patients** | **Controls** | **Patients** | **Controls** |  |
| Reijneveld et al 2001[46] | - Anticonvulsant medication^a^ - Epileptic seizures^a^ | n.d. | - Handedness (CPS)^a^ - KPS^a^ - Barthel Index^a^ - NFSS^a^ | - Handedness (CPS): right 96% | Not specified n=3 |
| Ruge et al 2010[47] | - Mild transient weakness of the left hand 3% - Seizures 64% - Anticonvulsive medication 55% - Generalised seizures 39% - 1+ seizure-related event 36% - Focal onset of seizures 24% - Recurrent headache, vertigo or tinnitus | n.d. | - KPS: mean 83.6 (6.5) | n.d. | Not meeting histological inclusion criteria n=3 |
| Bizzi et al 2012[48] | n.d. | n.d. | n.d. | - Edinburgh handedness inventory: right 100% | - |
| Mattavelli et al 2012[49] | n.d. | n.d. | n.d. | n.d. | - |
| Mu et al 2012[50] | - Use of AEDs 91% - Seizures 82% - Syncope, dizziness or headache 18%   Mannitol treatment 9% | n.d. | - Handedness: right-handed 100% - SDS: mean 39.36 (7.187) - SAS: mean 36.55 (11.19) - WAIS-Revised in China (n.d.) - VIQ: mean 102.5 (13.7) t=-0.236, p=0.819 - PIQ: mean 99.96 (13.94) t=-1.29, p=0.229 | - SDS 41.27 (5.312) - SAS 38.64 (6.975) - VIQ 102.8 (13.86) - PIQ 104.8 (15.42) | - |
| Plaza et al 2013[51] | n.d. | n.d. | - Handedness: left 40%; right 60% | - Handedness: left 40%; right 60% | - |
| Satoer et al 2013[44] *and* 2018[45] | n.d. | n.d. | - Handedness: left 7.4% | - Handedness: left 9.5% | - |
| Habets et al 2014[52] | - Epilepsy 50% - Neurological deficits 48% - Headache 31% - AEDs 55% - Corticosteroids 65% | n.d. | n.d. | n.d. | Fatigue, visual or dysphasic disorder, emotional disturbances, and/or time constraints n=18 |
| Huang et al 2014[53] | - n.d. | n.d. | Handedness: right 100% | - Handedness: right 100% | - |
| Kinno et al 2014[54] | Seizures | n.d. | - Edinburgh Handedness Inventory | n.d. | - |
| Antonsson et al 2018[55] | Seizures 70% | n.d. | - Narrative writing task and spontaneous speech task (n.d.) - Handedness: right 91%; left 9% | n.d. | Not meeting histological inclusion criteria n=1  Inflammation n=1 |
| De Witte et al 2018[56] | n.d. | n.d. | - Edinburgh Handedness Inventory: right 100% | n.d. | - |
| Zhang et al 2018[57] | n.d. | n.d. | - Edinburgh Handedness Inventory: right 100% - MRCS grade V - KPS: median 100 (80–100) | - Edinburgh Handedness Inventory: right 100% - KPS: median 100 (100) | Superfluous head movement  Patients n=7  Controls n=3 |
| Hu et al 2020[58] | n.d. | n.d. | - Edinburgh Handedness Inventory: right-handed 100% | - Edinburgh Handedness Inventory: right 100% | Not specified n=10 |
| Mooijman et al 2022[59] | n.d. | n.d. | - Handedness: right 78%; left 22% | n.d. | Recurrent tumour n=10  Not specified n=2  Co-morbidities n=2 |
| Tarantino et al 2022[60] | n.d. | n.d. | - Edinburgh Handedness Inventory: right 91%; bilateral 9% | - Edinburgh Handedness Inventory: right 95%; left 5% | Severe sustained attention deficit, low compliance or fatigue n=3 |
| Wang et al 2022[61] | - Charlson comorbidity index: 0–1 72%; 2–3 27%; >4 1% | Comorbidity score | - KPS: 80–100 98%, 50–70 2% | Age-adjusted Charlson comorbidity index | Not meeting histological inclusion criteria n=5 |

^a^ Demographics reported for full sample only; not reported for sample without excluded patients

NFSS: Neurologic Functional Status Scale; KPS: Karnofsky Performance Scale; CPS: ; SDS: Self-Rating Depression Scale; SAS: Self-Rating Anxiety Scale; VIQ: ; PIQ: ; MRCS: Medical Research Council Scale for Muscle Strength; HADS: German version hospital anxiety and depression scale; WAIS: Wechsler Adult Intelligence Scale; WISC: Wechsler Intelligence Scale for Children; HRQoL: Health-related quality of life; EORTC: The European Organization for Research and Treatment of Cancer; QLQ: Quality of Life questionnaire; GOS: Glasgow Outcome Scale; AcroQoL: The Acromegaly Quality of Life Questionnaire; WIE: ; PRL: ; STAI: State-Trait Anxiety Inventory; ICARS: ; FAR: Facial Affect Recognition; ToM: Theory of Mind; SST: Strange Stories Test; CBCL: Child Behavior Checklist; TRF: Teacher Report Form; BRIEF: Behavior Rating Inventory of Executive Function; CRT: Chemo-radiotherapy; AVLT: Auditory Verbal Learning Test; WMS: Wechsler Memory Scales; RMT: Recognition Memory Test; SCOLP: Speed and Comprehension of Language Processing; TMT: Trail-Making Test; AVLT: Auditory Verbal Learning Test; MMSE: Mini Mental State Examination; VFT: Verbal Fluency Test; BNT: Boston Naming Test; SDMT: Symbol-Digit Modalities Test; BLAST: Brief Language Assessment for Surgical Tumor Patients; AAT: Aachener Aphasie Test; WISC: Wechsler Intelligence Scale for Children; CMS: Children’s Memory Scale; TAP: Test of Attention Performance; CPT: Conner’s Continuous Performance Test; DSST: Digit-Symbol Substitution Test; DST: Digit Span Test; VLMT: Verbal Learning and Memory Test; VVLT: Visual Verbal Learning Test; MoCA: Montreal Cognitive Assessment; Wechsler Adult Intelligence Scale; AMIPB: Adult Memory and Information Processing Battery; LMW-R: Luria’s Memory Words Test – Revised; COWAT: Controlled Oral Word Association Test; MVGT: Munchner Verbaler Gedachtnistest; CANTAB: Cambridge Neuropsychological Test Automated Battery; RWT: Regensburger Verbal Fluency; BeSS: Bedomning av subtila sprakstorningar; SRP: Selective Remining Procedure; MDT: Memory Distractor Test; AQ: Aphasia Quotient; ABC: Aphasia Battery for Chinese Speakers; BADS: Behavioural Assessment of the Dysexecutive Syndrome for Children; CAMCOG: Cambridge Cognition Examination; LDMT: Letter Digit Modalities Test; BDAE: Boston Diagnostic Aphasia Examination Reading Sentences and Paragraphs Test

n.s. Not significant

n.d. No data reported

(References refer to references in main report)
