# Supplementary material for: Can cognitive function tests discriminate between patients with glioma and healthy controls prior to treatment? A systematic review
Source: PLoS One. 2025 Aug 6;20(8):e0329663. doi: 10.1371/journal.pone.0329663 (PMC12327679; doi:10.1371/journal.pone.0329663)
Supplement: S6 Table — (DOCX) [file pone.0329663.s006.docx]

S6 Table. Summary of studies that include patients with frontal or temporal location of glioma in systematic review

| **Study** | **Patients** | | **Controls** | **Cognitive Testing** | | | | | |
| --- | --- | --- | --- | --- | --- | --- | --- | --- | --- |
|  |  |  |  | **Cognitive Function** | **Measure** | **Mean (SD)** | | **Significance Testing** | **Effect size** |
|  | **N** | **Tumour Location**  **%** | **N** |  |  | **Patients** | **Controls** |  | **(Hedges’ *g*)** |
| Bizzi et al 2012[48] | n=19 | Left frontal 100%  VLPM^a^ 42%  VLPC^a^ 58% | n=10 | Language | AAT* Written Language | VLPM: 88.9 (1.4)  VLPC: 81.0 (16.0) | 90 (0) | VLPM: p=.083  VLPC: p=.007 | VLPM: -1.09  VLPC: -0.76 |
|  |  |  |  |  | AAT* Comprehension | VLPM: 113.2 (11.1)  VLPC: 114.6 (5.0) | 119.0 (1.3) | VLPM: p=.203  VLPC: p=.011 | VLPM: -0.72  VLPC: -1.15 |
|  |  |  |  |  | AAT* Communicative Behaviour | VLPM: 4.9 (.4)  VLPC: 4.1 (1.1) | 5 (0) | VLPM: p=.696  VLPC: p=.021 | VLPM: -0.35  VLPC: -1.11 |
|  |  |  |  |  | AAT* Articulation and Prosody | VLPM: 4.9 (.4)  VLPC: 3.9 (.9) | 5 (0) | VLPM: p=.694  VLPC: p=.002 | VLPM: -0.35  VLPC: -1.66 |
|  |  |  |  |  | AAT* Phonological Structure | VLPM: 4.9 (.4)  VLPC: 4.2 (.9) | 5 (0) | VLPM: p=.696  VLPC: p=.021 | VLPM: -0.35  VLPC: -1.20 |
|  |  |  |  |  | Token Test | VLPM: 45.5 (10.4)  VLPC: 44.5 (5.6) | 49.1 (1) | VLPM: p=.762  VLPC: p=.004 | VLPM: -0.48  VLPC: -1.10 |
|  |  |  |  |  | VFT* Semantic | VLPM: 39.6 (12.1)  VLPC: 33.3 (13.7) | 50.7 (9.4) | VLPM: p=.083  VLPC: p=.002 | VLPM: -0.99  VLPC: -1.42 |
|  |  |  |  |  | VFT* Phonemic | VLPM: 32.5 (10.6)  VLPC: 18.5 (10.9) | 44.7 (11.3) | VLPM: p=.055  VLPC: p=.000 | VLPM: -1.06  VLPC: -2.26 |
| Mattavelli et al 2012[49] | n=22 | Left frontal 100% | n=26 | Decision-making | Gambling Task: Good | 27.72 (13.69) | 43 (22.74) | p=.008 | -0.78 |
|  |  |  |  |  | Gambling Task: Bad 1 | 33.32 (13.07) | 30.46 (17.31) | p=.53 | 0.18 |
|  |  |  |  |  | Gambling Task: Neutral | 20.95 (7.94) | 13.23 (7.15) | p=.001 | 1.01 |
|  |  |  |  |  | Gambling Task: Bad 2 | 18 (6.71) | 13.30 (6.5) | p=.018 | 0.70 |
|  |  |  |  |  | Gambling Task: Reaction Time | n.d. | n.d. | p=.003 | - |
| Mu et al 2012[50] | n=11 | Left frontal 100% | n=11 | Multiple cognitive functions | DST* Total | 10.18 (2.316) | 13.91 (2.587) | p=.000 | -1.46 |
|  |  |  |  |  | DST* Forwards | 6.36 (1.567) | 8.18 (1.079) | p=.016 | -1.30 |
|  |  |  |  |  | DST* Backwards | 3.91 (1.375) | 5.73 (1.954) | p=.003 | -1.04 |
|  |  |  |  | Memory | Tapping Test Total | 14.64 (2.501) | 16.82 (2.04) | p=.079 | -0.92 |
|  |  |  |  |  | Tapping Test Forwards | 7.73 (1.348) | 9 (1.265) | p=.067 | -0.93 |
|  |  |  |  |  | Tapping Test Backwards | 6.91 (1.64) | 7.82 (1.25) | p=.194 | -0.60 |
|  |  |  |  | Executive function | Modified Card Sorting Test: Category Control | 3.27 (1.737) | 4.27 (.905) | p=.128 | -0.69 |
|  |  |  |  |  | Modified Card Sorting Test: Preservative Response | 6.18 (4.119) | 2.73 (3.101) | p=.047 | 0.91 |
|  |  |  |  |  | Modified Card Sorting Test: Failure to Maintain a Set | 1.55 (1.036) | 1.27 (1.489) | p=.615 | 0.21 |
|  |  |  |  |  | Modified Card Sorting Test: Preservative Error | 1.82 (1.537) | 0.73 (.905) | p=.082 | 0.83 |
|  |  |  |  |  | Modified Card Sorting Test: Number of Answers to Complete First Category | 11.91 (10.58) | 9.18 (3.188) | p=.574 | 0.34 |
|  |  |  |  |  | Modified Card Sorting Test: % Preservative Response | 40.91 (21.72) | 53.41 (11.31) | p=.128 | -0.69 |
|  |  |  |  |  | Modified Card Sorting Test: Total Errors | 18.73 (6.405) | 14.82 (3.763) | p=.061 | 0.72 |
| Huang et al 2014[53] | n=12 | Left frontal 58%  Right frontal 42% | n=12 | Multiple cognitive functions | MoCA* | 20.2 (1.5) | 27.9 (1.1) | p<.01 | -5.65 |
| Kinno et al 2014[54] | n=21 | Left frontal 100%  LPMC^g^ 33%  F3^g^ 33%  Other left frontal regions 33% | n=28 | Language | Picture-Sentence Matching Task: Error Rates Active | 8.13 | 3.1 | p<.0001 | - |
|  |  |  |  |  | Picture-Sentence Matching Task: Error Rates Passive | 11.82 | 2.9 | p<.0001 | - |
|  |  |  |  |  | Picture-Sentence Matching Task: Error Rates Scrambled | 22.34 | 2.2 | p<.0001 | - |
|  |  |  |  |  | Picture-Sentence Matching Task: Error Rates One-Argument | 2.4 | 1.8 | p=.76 | - |
|  |  |  |  |  | Picture-Sentence Matching Task: Error Rates Control Test | 2.32 | 2.2 | n.d. | - |
|  |  |  |  |  | Picture-Sentence Matching Task: Reaction Times (ms) Active | 3334 | 2052 | n.d. | - |
|  |  |  |  |  | Picture-Sentence Matching Task: Reaction Times (ms) Passive | 3399 | 3129 | n.d. | - |
|  |  |  |  |  | Picture-Sentence Matching Task: Reaction Times (ms) Scrambled | 3499 | 3242 | n.d. | - |
|  |  |  |  |  | Picture-Sentence Matching Task: Reaction Times (ms) One-Argument | 2760 | 2644 | n.d. | - |
|  |  |  |  |  | Picture-Sentence Matching Task: Reaction Times (ms) Control Test | 2756 | 2766 | n.d. | - |
| Hu et al 2020[58] | n=17 | Temporal lobe 100% | n=28 | Multiple cognitive functions | DST* Total | 8.44 (3.09) | 11 (2.67) | p=.518 | -0.89 |
|  |  |  |  |  | DSST* | 7.4 (2.88) | 11.88 (1.64) | p=.028 | -2.01 |
|  |  |  |  |  | Arithmetic § | 5.89 (2.42) | 10.63 (2) | p=.007 | -2.15 |
|  |  |  |  | Memory | Memory test§ | 6.43 (4.72) | 11.88 (1.55) | p=.063 | -1.71 |
|  |  |  |  | Language | WAIS* Similarities Test | 6.38 (2.88) | 10 (1.07) | p=.035 | -1.82 |
|  |  |  |  | Visuospatial function | Mapping§ | 5.63 (2.56) | 9.88 (.64) | p=.000 | -2.54 |
|  |  |  |  |  | Visuospatial test § | 7 (3.87) | 10.63 (1.6) | p=.021 | -1.33 |

^a^ Anterior ventrolateral premotor (VLPM); posterior ventrolateral precentral (VLPC)

* AMIPB: Adult Memory and Information Processing Battery; DST: Digit Span Test; MoCA: Montreal Cognitive Assessment; WAIS: Wechsler Adult Intelligence Scale; AAT: Aachener Aphasie Test; VFT: Verbal Fluency Task; DSST: Digit-Symbol Substitution Test

n.s. Not specified

n.d. No data reported

(References refer to references in main report)
