# Supplementary material for: Can cognitive function tests discriminate between patients with glioma and healthy controls prior to treatment? A systematic review
Source: PLoS One. 2025 Aug 6;20(8):e0329663. doi: 10.1371/journal.pone.0329663 (PMC12327679; doi:10.1371/journal.pone.0329663)
Supplement: S7 Table — (DOCX) [file pone.0329663.s007.docx]

S7 Table. Matrix showing which cognitive function was tested in which studies with only frontal or temporal lobe glioma and whether a significant difference was found between patients and control groups

| **Study** | **Tumour Location** | **Control Group Condition** |  | **Cognitive Function** | | | | | | |
| --- | --- | --- | --- | --- | --- | --- | --- | --- | --- | --- |
| **GLIOMA** |  |  | **Multiple Cognitive Functions** | **Language** | **Memory** | **Information Processing** | **Executive Function** | **Decision-Making** | **Attention** | **Visuospatial Function** |
| Bizzi et al 2012[48] | Frontal | Healthy |  | ↓↓↓↓↓↓↓↓ |  |  |  |  |  |  |
| Mattavelli et al 2012[49] | Frontal | Healthy |  |  |  |  |  | ↓↓↓↓  • |  |  |
| Mu et al 2012[50] | Frontal | Healthy | ↓↓↓ |  | ••• |  | ↓  •••••• |  |  |  |
| Huang et al 2014[53] | Frontal | Healthy | ↓ |  |  |  |  |  |  |  |
| Kinno et al 2014[54] | Frontal | Healthy |  | ↓↓↓  •  °°°°°° |  |  |  |  |  |  |
| Hu et al 2020[58] | Temporal | Healthy | ↓↓  • | ↓ | • |  |  |  |  | ↓↓ |
