# Supplementary material for: Can cognitive function tests discriminate between patients with glioma and healthy controls prior to treatment? A systematic review
Source: PLoS One. 2025 Aug 6;20(8):e0329663. doi: 10.1371/journal.pone.0329663 (PMC12327679; doi:10.1371/journal.pone.0329663)
Supplement: S9 File — (PDF) [file pone.0329663.s009.pdf]

Systematic Review Protocol

Title

Can cognitive function tests discriminate between patients with and without a brain tumour prior to treatment?: A systematic review

This report is a protocol of a systematic review developed according to Preferred Reporting Items for Systematic review and Meta-Analysis Protocols (PRISMA-P) 2015 (Moher et al., 2015).

Authors

Laura Standen, Queen Mary University of London, l.standen@qmul.ac.uk, Centre for Prevention, Detection and Diagnosis, Wolfson Institute of Population Health.

Professor Suzanne Scott, Queen Mary University of London, suzanne.scott@qmul.ac.uk, Centre for Prevention, Detection and Diagnosis, Wolfson Institute of Population Health.

Professor Fiona Walter, Queen Mary University of London, fiona.walter@qmul.ac.uk, Wolfson Institute of Population Health.

Competing Interests

The authors do not have any competing interests to declare.

Funding Sources

Barts Charity

Introduction

Background

Primary brain tumours are experienced by approximately 7 per 100,000 in the UK population (McNamara, 2011). Between 1995 and 2015, there were 81,135 primary brain tumours diagnosed in England, of which 19.5k diagnoses were in people aged 30–54 and 52k diagnoses were in people aged 55+ (Philips et al., 2018). Brain tumour prevalence has been rising over the past few decades (de Vocht, 2016); although, this might not be an increase in occurrence, but rather improved diagnostic tools and clinical approaches (Walsh et al., 2014). Brain cancer has a low 5-year survival rate, and patients often have severe physical and

psychological deficits following treatment (Loizidou et al., 2022). Overall, survival rates for brain tumours for 1 year are approximately 40% and for 5 years are approximately 12% (Office for National Statistics, 2018), while more aggressive types of brain tumour can have even shorter survival rates (glioblastoma multiforme median survival rate of 6.1 months (Philips et al., 2018)). A large proportion (62%) are diagnosed through emergency services (Fraulob & Davies, 2020), which is generally associated with much worse survival outcomes (Penfold et al., 2017). Evidence indicates that mortality rates for brain tumours have not improved significantly alongside advances in treatment over the last 30 years (Gould, 2018; Philips et al., 2018). Further research focusing on brain tumours is a high priority due to these poor survival outcomes (de Vocht, 2016; Hamilton et al., 2022), and research identifying early symptoms of brain tumours is supported by the National Institute for Health and Care Excellence (NICE, 2021), the National Institute for Health and Care Research (NIHR, 2019), the Cancer Research UK (2022) research strategy, and the Tessa Jowell Brain Cancer Mission (2019), with the aim of improving survival rates, patient outcomes and quality of life associated with earlier detection and diagnosis. Furthermore, as the Covid-19 pandemic has placed a massive burden on resources and accessibility of healthcare services in the UK, this further highlights the importance of more timely diagnosis of brain tumours at a less resource-demanding stage (Hamilton et al., 2022).

Implications of prolonged time to diagnosis

A recent systematic review has indicated that the average time from noticing the first symptom to receiving a diagnosis for a brain tumour is a minimum of 14 weeks (Ghandour et al., 2021). An advanced-stage diagnosis or even a complete lack of diagnosis severely impacts patient outcomes and survival for brain tumours (Senan et al., 2022), and it is well evidenced that cancer survival is fundamentally dependent on the stage at which the cancer is diagnosed (Hamilton et al., 2022). Most malignant brain tumours are terminal without timely diagnosis (Ghandour et al., 2021), and brain tumours diagnosed at an advanced stage are also associated with higher neurocognitive deficits due to the need for more extensive and intrusive surgical treatments, thus negatively impacting quality of life (Penfold et al., 2017). Prompt diagnosis of brain tumours could facilitate treatment and improve survival outcomes with as few condition-associated deficits as possible (Grant et al., 2020; Khalil et al., 2021). Furthermore, a prolonged diagnosis pathway that involves multiple visits to a GP and initial misdiagnosis, due to vague or non-specific symptoms as well as underreporting of brain tumour-associated symptoms, can cause higher levels of stress and anxiety for patients (Christensen & Huniche,

2020). Research has indicated that only 1% of brain tumour patients are currently referred via the two-week-wait pathway (Walter et al., 2019), which is intended to expedite the cancer diagnostic pathway, therefore aiming to reduce anxiety and stress experienced by patients (Salander et al., 1999). Furthermore, one-third of patients visit their GP three or more times before receiving a referral (Ozawa et al., 2018; Zienius et al., 2019), and one-third of GPs feel there are avoidable delays during the pathway to referral (Ozawa et al., 2018).

Research in brain tumour diagnosis has been overlooked in the past, and it is important to note that there are some concerns that research into improving diagnosis rates would be wasted due to how incredibly difficult it can be to identify early symptoms of brain tumours (Penfold et al., 2017). In addition, more timely diagnosis research is challenging because different types of brain tumour have varying symptoms, providing a challenge to identify a wide spectrum of symptoms across a number of different diagnoses (Penfold et al., 2017). Advancements in diagnostic imaging for brain tumours have not been accompanied by advancements in treatments that are impacting overall survival or outcomes (Wilkinson, 2005). Brain cancer currently has limited treatment options available, with survival rates remaining low despite treatment improvements, and the focus for healthcare professionals is often on quality of life rather than survival outcomes (McNamara, 2011). However, patients who experience seizures as a symptom can report better prognosis outcomes than patients who do not experience seizures; this is possibly because seizures are a much clearer indication of a brain tumour, which therefore facilitates earlier diagnosis (McNamara, 2011). More research will clarify the extent to which a more timely diagnosis of brain tumours can improve patient outcomes and survival, particularly as treatments continue to be improved; however, importantly, more timely diagnosis has the potential to improve patient experience and quality of life (Lyratzopoulos et al., 2014).

Symptoms prior to diagnosis

The majority of patients for whom imaging reveals a brain tumour often present with seizure symptoms or neurological symptoms (Kracht & Heiss, 2014), including headache; behavioural / cognitive changes; personality changes; seizures; speech issues; sensory issues; focal neurology (including stroke); fits, faints, and falls; weakness; confusion; memory loss; fatigue; non-specific neurological; and general non-specific symptoms (Ozawa et al., 2018; Perkins & Liu, 2016). Subtle cognitive impairments can be present for months prior to a diagnosis (Fraulob & Davies, 2020; Scott et al., 2019; Walter et al., 2019), and these symptoms can impact detrimentally on patients' psychological and physical wellbeing, as well as quality of

life (Fraulob & Davies, 2020). Only one-quarter (27%) of patients are referred for imaging on their first visit to a GP; a further one-quarter (27%) are referred within their second or third visit to a GP; and over 2 in 5 (43%) patients visited a GP more than 3 times before referral (Sage et al., 2019). Patients with non-specific symptoms, such as headache and cognitive / behavioural deficits, often experience the longest time from noticing symptoms to being referred (Zienius et al., 2019), and patients with headache, behavioural / cognitive changes (in particular, memory loss), and those with non-specific symptoms are more likely to have attended a GP three or more times before referral (Ozawa et al., 2018). This is likely to be impacted by the under-reporting by patients of these often vague and non-specific symptoms as well (Scott et al., 2019). Case-control studies have indicated that the strongest positive predictive values for combined symptoms are for headache with cognitive deficit symptoms (7.2%) and cognitive deficit symptoms alongside weakness (9.6%) (Ozawa et al., 2019). This highlights that early cognitive symptoms could alert GPs to consider referring a patient for neuro-imaging, although the under-reporting of these symptoms remains a challenge (Walter et al., 2019). Identifying cognitive deficit symptoms could help triage patients and provide the rationale for GP referral (Zienius et al., 2019), potentially leading to more timely diagnosis. However there is a need for tools to assist GPs to identifying cognitive deficits when deciding which patients to refer for neuro-imaging (Ozawa et al., 2019).

Routes to diagnosis / challenges in primary care

Cognitive deficits are not mutually exclusive to diagnosis of a brain tumour: they can be signs of dementia or Alzheimer's disease (Perkins & Liu, 2016) as well as stroke (Ozawa et al., 2018), multiple sclerosis (Halabchi et al., 2017), migraine (Vuralli et al., 2018), and other conditions, including no medical condition (Smith, 2002). This increases the challenge of identifying cognitive deficits associated with brain tumours, but it also presents a challenge for the GP to identify cognitive symptoms specifically associated with brain tumours (Salander et al., 1999), therefore knowing which patients require an urgent referral for neuro-imaging. The recommended route to diagnosis for brain tumours is for a patient with symptoms to attend primary care for a GP assessment, and then to be referred for neuro-imaging within the two-week-wait cancer diagnostic pathway (NICE, 2015). However, research has suggested that the two-week-wait pathway is ineffective for patients with brain tumours, and that the majority of patients are still diagnosed via alternative, often emergency, pathways (Ceronie et al., 2021). Research has further suggested that among symptomatic referrals for neuro-imaging, only 1% of patients are diagnosed with a brain tumour, and neuro-imaging has indicated no evidence of

any neurological condition in nearly 80% of patients referred (Zienius et al., 2019). In addition, the two-week-wait referral pathway has the lowest use for patients with brain tumours (6%) across all cancer sites (Zhou et al., 2018), and this is possibly due to a lack of clear referral guidelines and diagnostic support tools for often vague and non-specific symptoms associated with brain tumours (Ceronie et al., 2021).

Due to the subtle nature of the cognitive symptoms associated with brain tumours, patients are also less likely to report the symptom to a GP (Salander et al., 1999), adding further challenge for GPs to identify which patients to refer. Furthermore, previous research has indicated that retrospectively family and friends quite often notice subtle cognitive / behavioural changes in the patient, but that these go unmentioned to the GP (Ozawa et al., 2018). The number of patients diagnosed with brain tumour per GP is low; statistically a patient is less likely to have a brain tumour than another condition, which leads GPs to investigate other possible causes before considering brain tumour referral (Penfold et al., 2017). More effective measures and assessments of cognitive symptoms, which are often subtle and / or non-specific at initial GP presentation, could facilitate more timely diagnosis (Jones et al., 2021), by guiding GPs when deciding whether to refer patients for neuro-imaging (Ozawa et al., 2019). Referring everyone who presents with a headache or subtle cognitive symptoms for neuro-imaging would add unnecessary stress to patients (particularly those who do not have a brain tumour), increase the risk of false positives or of incidental findings (e.g. non-pathological aneurysms), overwhelm the already burdened neuro-imaging service, and increase healthcare costs (Kostopoulou et al., 2019; Mohammad et al., 2016; Walter et al., 2019; Zienius et al., 2019). A primary care triage support tool for subtle cognitive symptoms could be crucial innovation to avoid over-referral. It is fundamental that GPs proactively raise potential cognitive symptoms with patients and their families, with the support of triage tools, in order to improve diagnosis rates (Ozawa et al., 2019).

Measuring cognitive function

Cognitive function tests are used to identify subtle cognitive deficits in executive function and are typically comprised of a series of tasks for the patient to complete, which are then measured against validated cut-off scores (Ciesielska et al., 2016). In secondary care settings, cognitive function tests are used to monitor brain tumour progression, recurrence, and specific areas of function deficit, and in particular are used post-surgically to understand the focus for neurocognitive rehabilitation (Becker et al., 2016; Robinson et al., 2015). Cognitive screening within secondary care is often a short measure, while cognitive test batteries are much longer

and involve more in-depth, all-encompassing neurocognitive assessments. There is currently no gold standard cognitive function test for brain tumour patients (Renovanz et al., 2018), though recommended practice is a neurocognitive test battery that lasts a minimum of 1 hour (Becker et al., 2016). As such, cognitive function tests can be long and arduous for patients, therefore reducing the accessibility and feasibility of these tests (Renovanz et al., 2018), particularly as a tool to be used within primary care. Cognitive tests include, among others, the clock-drawing test (CDT), Montreal cognitive assessment (MoCA), mini-mental state exam (MMSE), abbreviated mental test (AMT), memory impairment screen (MIS), mental status questionnaire (MSQ), and the short portable mental status questionnaire (SPMSQ) (Janssen et al., 2017). The Neuropsychiatric Inventory Questionnaire is used to assess personality and behavioural changes (Janssen et al., 2017), and other cognitive tests include the mini Examen Cognoscitivo (MEC – Spanish), the Eurotest, the Fototest, and the memory alteration test (M@T) (Carnero-Pardo et al., 2022). The MSQ, SPMSQ, MIS, AMT and the MMSE are used commonly to identify mild cognitive impairments often associated with dementia (Janssen et al., 2017). As such, it is important to identify cognitive function tests that have been studied in those with brain tumours.

Currently, the evidence is conflicting over the sensitivity of shorter cognitive assessment tools for brain tumour patients; one recent within-patient comparison study (Robinson et al., 2015) found that the Montreal cognitive assessment (MoCA) used as a shorter assessment in secondary care with brain tumour patients post-surgical treatment had a much lower sensitivity when compared within-group with a comprehensive cognitive assessment that lasted 1–1.5 hours (30.4% vs 69.6% identification of cognitive deficit respectively). However, the MoCA and its recommended cut-off score (26) was developed using samples comprised of patients with memory loss and Alzheimer’s disease (Nasreddine et al., 2005). It is possible that a modified cut-off score or other cognitive function tests tested in brain tumour patient populations could have higher sensitivity. In secondary care, cognitive function tests can be chosen that assess only one particular cognitive function, based on the location of the tumour and the deficits that the patient is likely to experience (Robinson et al., 2015). However, full neurocognitive test batteries that assess for all executive function can last anywhere between 1 and 8 hours (Robinson et al., 2015), which is not feasible for diagnostic application within the primary care setting. Therefore, it is crucial to identify whether individual cognitive function tests have sufficient sensitivity to identify a brain tumour in patients presenting with a wide possible range of cognitive impairment.

In sum, if cognitive deficits can highlight a possible brain tumour (Renovanz et al., 2018) cognitive function tests could be an important way to identify which patients should be referred as a priority for neuro-imaging to assess for a neurological condition. Many patients present to primary care with a headache, as well as cognitive deficits, which can be associated with brain tumours or other neurological conditions, and it is a challenge for GPs to identify which patients should be referred for imaging. Cognitive deficit symptoms are often underreported within primary care or go unrecognised as symptoms, and this detrimentally impacts the patient experience of the diagnostic pathway for brain tumours. Cognitive function tests could be a useful triaging tool within primary care to facilitate the referral pathway, therefore reducing the burden on patients and their quality of life, and alongside potential future improvements in brain tumour treatment, could improve patient outcomes and survival. However, it is not yet clear which cognitive function tests can discriminate between patients with and without a brain tumour, and furthermore, if these tests are suitable within the primary care setting where time and resources are limited. This review is fundamental to assess currently used cognitive function tests in identifying a brain tumour.

Objectives

This systematic review aims to determine if cognitive function tests can discriminate between patients with and without a brain tumour prior to diagnosis.

Methods

Inclusion criteria

We will include studies of patients with a brain tumour (defined as an abnormal growth in the brain; diagnosed via radiography – e.g. MRI or CT (McFaline-Figueroa & Lee, 2018) – from any race, gender, ethnicity, or socioeconomic background. We will only include participants pre-treatment as brain tumour treatment can often result in further or worsening cognitive deficits (Coomans et al., 2019). We will include sub-groups where relevant for analysis, including age bracketing, that might be present in the reviewed studies, as well as education levels and gender, as there has been some evidence that these factors can influence the outcomes of cognitive function tests (Chan et al., 2020; Villalobos et al., 2022). We will include all study designs that include patients with a brain tumour compared with a control comparison group. We will include all studies that use a cognitive function test (task-based assessment of neurological executive function) to assess cognitive deficits with regards to identifying a brain

tumour. We will include studies in all languages, from all timeframes, and with all publication statuses.

Exclusion criteria

We will exclude any studies that include patients who are post-treatment. We will exclude studies that include patients who have a metastatic brain cancer diagnosis as the scope of this review is only within primary brain tumours. We will exclude studies that do not have a control comparison group, as this will not allow for analysis of discrimination between participants with and without brain tumour.

Information sources

Studies will be identified by searching the following electronic databases: Medline (via PubMed), CENTRAL, and Embase; as well as searching reference lists in studies included in the review, grey literature, trials registers (e.g. ClinicalTrials.gov and WHO ICTRP), clinical study reports, dissertations, and conference abstracts and proceedings.

Search strategy

The full search strategy can be seen in the appendices. As an example, we will use the following search strategy within the PubMed database: ((Brain tumour*[Title/Abstract]) OR (brain cancer*[Title/Abstract]) OR (brain neoplasms[MeSH Terms])) AND ((Cognit* function* test*[Title/Abstract]) OR (cognit* function* assessment*[Title/Abstract]) OR (cognitive function* exam*[Title/Abstract]) OR (executive function* test*[Title/Abstract]) OR (executive function* assessment*[Title/Abstract]) OR (executive function* exam*[Title/Abstract]) OR (neuropsycholog* assessment*[Title/Abstract]) OR (neuro-psycholog* assessment*[Title/Abstract]) OR (neuropsycholog* exam*[Title/Abstract]) OR (neuro-psycholog* exam*[Title/Abstract]) OR (cognit* test*[Title/Abstract]) OR (cognit* assessment*[Title/Abstract]) OR (cognit* exam*[Title/Abstract]) OR (cognit* abilit* test*[Title/Abstract]) OR (clock-drawing test[Title/Abstract]) OR (Montreal cognitive test[Title/Abstract]) OR (mini-mental state exam[Title/Abstract]) OR (abbreviated mental test[Title/Abstract]) OR (memory impairment screen[Title/Abstract]) OR (mental status questionnaire[Title/Abstract]) OR (short portable mental status questionnaire[Title/Abstract]) OR (neuropsychiatric inventory questionnaire[Title/Abstract]) OR (mini examen cognoscitivo[Title/Abstract]) OR (Eurotest[Title/Abstract]) OR (Fototest[Title/Abstract]) OR (memory alteration test[Title/Abstract]) OR (verbal fluency[Title/Abstract]) OR

(memory[Title/Abstract]) OR (mental capacity[Title/Abstract]) OR (Neuropsychological tests[MeSH Terms])).

Study records

Two authors will independently assess all abstracts identified by the study search for screening, eligibility, inclusion and exclusion in the review. Once this has been completed, we will discuss together the studies to be included in the review, and any disagreements will be taken to a third author for decision.

Data extraction and management

Data will be extracted independently by two authors from all studies that meet the inclusion criteria. Data collected will include participant demographics; recruitment methods; diagnosis data, including tumour type and stage where possible, for the brain tumour population group; the cognitive function tests used and how they were administered; the study design and methods; study completion and drop-out rates; the study outcomes and results; and author conflicts of interest where relevant. Where data is missing, incomplete, or unclear, we will aim to contact the study authors for clarification where possible; where not possible, we will exclude any missing or incomplete data from the meta-analysis, but we will include it in the narrative synthesis.

Outcomes and Prioritisation

Primary outcome

- Comparison of cognitive function test outcomes in those with and without brain tumour
- Discrimination between participants with and without a brain tumour

Secondary outcomes

- Details of what each test entailed and how it was carried out
- Adverse effects experienced by participants as a result of carrying out the cognitive function test

Risk of Bias in Individual Studies

We will assess for risk of bias in study methods and outcomes by using the Quality Assessment of Diagnostic Accuracy Studies (QUADAS-2), as a widely recognised and recommended tool

(Huang & Huang, 2022), which assesses four domains: (1) patient selection, (2) index test(s), (3) reference standard, and (4) flow and timing. This assessment of risk of bias will be carried out independently by two authors and then reviewed together. Any disagreements about risk of bias for studies to be included in the review will be taken to a third author. The risk of bias will be identified for each study clearly within the review, and will be considered and outlined as part of the data synthesis.

Data synthesis

We will collate all data and provide a narrative synthesis that will detail the population in the study, the cognitive function test(s) used (including method and administration), adverse effects experienced by participants, and the results / outcomes of the test. We will include sub-group analyses where possible for bracketed age groups, education level, and gender.

Where studies have used the same cognitive test(s) and have used the same methods and administration of the test, and the same analysis of the data, a meta-analysis will be used to collate all relevant data and to determine mean differences, 95% confidence intervals, and p values. A fixed-effect model will be used to determine degree of heterogeneity of the included studies. Where possible for homogenous studies, we will collate sensitivity, specificity, positive predictive values (PPV), and negative predictive values (NPV) data, and where relevant, diagnostic odds ratios and a summary receiver operating characteristic curve will be determined (Deeks, 2001). Any missing summary data will be determined by calculating the summary statistic from the individual sample data where possible; where not possible, this data will be excluded from the meta-analysis. We will include sub-group analyses where possible for bracketed age groups, education level, and gender. Risk of bias, including selective reporting, will be considered when assessing study data for inclusion in the meta-analysis; only if risk of bias is determined to be low will the study data be included in a meta-analysis. We will perform the meta-analysis using R Studio software.

The strength of the evidence will be assessed using the Grading Recommendations, Assessment, Development, and Evaluations (GRADE) framework (Schünemann et al., 2020). We will carry out a sensitivity analysis on any meta-analysis, and we will report this in the review.

References

Becker, J., Steinmann, E., Könemann, M., Gabske, S., Mehdorn, H. M., Synowitz, M., Hartwigsen, G., & Goebel, S. (2016). Cognitive screening in patients with intracranial tumors: Validation of the BCSE. *Journal of Neuro-Oncology*, *127*(3), 559–567. https://doi.org/10.1007/s11060-016-2064-6

Cancer Research UK. (2022). *Our Research Strategy*. https://www.cancerresearchuk.org/sites/default/files/cancer_research_uk_-_our_research_strategy.pdf

Carnero-Pardo, C., Rego-García, I., Mené Llorente, M., Alonso Ródenas, M., & Vílchez Carrillo, R. (2022). Utilidad diagnóstica de test cognitivos breves en el cribado de deterioro cognitivo. *Neurología*, *37*(6), 441–449. https://doi.org/10.1016/j.nrl.2019.05.007

Ceronie, B., Hart, T., Belete, D., Ramani, L., & Bahra, A. (2021). Isolated headache is not a reliable indicator for brain cancer: The 2-week wait pathway for suspected CNS malignancies. *Clinical Medicine*, *21*(6), e648–e655. https://doi.org/10.7861/clinmed.CM-2021-0223

Chan, J. Y. C., Wong, A., Yiu, B., Mok, H., Lam, P., Kwan, P., Chan, A., Mok, V. C. T., Tsoi, K. K. F., & Kwok, T. C. Y. (2020). Electronic Cognitive Screen Technology for Screening Older Adults With Dementia and Mild Cognitive Impairment in a Community Setting: Development and Validation Study. *Journal of Medical Internet Research*, *22*(12), e17332. https://doi.org/10.2196/17332

Christensen, H. M., & Huniche, L. (2020). Patient perspectives and experience on the diagnostic pathway of lung cancer: A qualitative study. *SAGE Open Medicine*, *8*, 2050312120918996. https://doi.org/10.1177/2050312120918996

Ciesielska, N., Sokołowski, R., Mazur, E., Podhorecka, M., Polak-Szabela, A., & Kędziora-Kornatowska, K. (2016). Is the Montreal Cognitive Assessment (MoCA) test better suited

than the Mini-Mental State Examination (MMSE) in mild cognitive impairment (MCI) detection among people aged over 60? Meta-analysis. *Psychiatria Polska*, *50*(5), 1039–1052. https://doi.org/10.12740/PP/45368

Coomans, M. B., van der Linden, S. D., Gehring, K., & Taphoorn, M. J. B. (2019). Treatment of cognitive deficits in brain tumour patients: Current status and future directions. *Current Opinion in Oncology*, *31*(6), 540–547. https://doi.org/10.1097/CCO.0000000000000581

de Vocht, F. (2016). Inferring the 1985–2014 impact of mobile phone use on selected brain cancer subtypes using Bayesian structural time series and synthetic controls. *Environment International*, *97*, 100–107. https://doi.org/10.1016/j.envint.2016.10.019

Deeks, J. J. (2001). Systematic reviews of evaluations of diagnostic and screening tests. *BMJ*, *323*(7305), 157–162. https://doi.org/10.1136/bmj.323.7305.157

Fraulob, I., & Davies, E. A. (2020). How do patients with malignant brain tumors experience general practice care and support? Qualitative analysis of English Cancer Patient Experience Survey (CPES) data. *Neuro-Oncology Practice*, *7*(3), 313–319. https://doi.org/10.1093/nop/npz062

Ghandour, F., Squassina, A., Karaky, R., Diab-Assaf, M., Fadda, P., & Pisanu, C. (2021). Presenting Psychiatric and Neurological Symptoms and Signs of Brain Tumors before Diagnosis: A Systematic Review. *Brain Sciences*, *11*(3), Article 3. https://doi.org/10.3390/brainsci11030301

Gould, J. (2018). Breaking down the epidemiology of brain cancer. *Nature*, *561*(7724), S40–S41. https://doi.org/10.1038/d41586-018-06704-7

Grant, R., Dowswell, T., Tomlinson, E., Brennan, P. M., Walter, F. M., Ben-Shlomo, Y., Hunt, D. W., Bulbeck, H., Kernohan, A., Robinson, T., & Lawrie, T. A. (2020). Interventions to reduce the time to diagnosis of brain tumours. *Cochrane Database of Systematic Reviews*, *9*. https://doi.org/10.1002/14651858.CD013564.pub2

Halabchi, F., Alizadeh, Z., Sahraian, M. A., & Abolhasani, M. (2017). Exercise prescription for patients with multiple sclerosis; potential benefits and practical recommendations. *BMC Neurology*, *17*(1), 185. https://doi.org/10.1186/s12883-017-0960-9

Hamilton, A. C., Donnelly, D. W., Fitzpatrick, D., & Coleman, H. G. (2022). Early-Onset Cancers in Adults: A Review of Epidemiology, Supportive Care Needs and Future Research Priorities. *Cancers*, *14*(16), Article 16. https://doi.org/10.3390/cancers14164021

Huang, Q.-X., & Huang, X.-W. (2022). QUADAS-2 tool for quality assessment in diagnostic meta-analysis. *Annals of Palliative Medicine*, *11*(5), Article 5. https://doi.org/10.21037/apm-22-204

Janssen, J., Koekkoek, P. S., Moll van Charante, E. P., Jaap Kappelle, L., Biessels, G. J., & Rutten, G. E. H. M. (2017). How to choose the most appropriate cognitive test to evaluate cognitive complaints in primary care. *BMC Family Practice*, *18*(1), 101. https://doi.org/10.1186/s12875-017-0675-4

Jones, O. T., Calanzani, N., Saji, S., Duffy, S. W., Emery, J., Hamilton, W., Singh, H., Wit, N. J. de, & Walter, F. M. (2021). Artificial Intelligence Techniques That May Be Applied to Primary Care Data to Facilitate Earlier Diagnosis of Cancer: Systematic Review. *Journal of Medical Internet Research*, *23*(3), e23483. https://doi.org/10.2196/23483

Khalil, A., Rahimi, A., Luthfi, A., Azizan, M. M., Satapathy, S. C., Hasikin, K., & Lai, K. W. (2021). Brain Tumour Temporal Monitoring of Interval Change Using Digital Image Subtraction Technique. *Frontiers in Public Health*, *9*. https://www.frontiersin.org/articles/10.3389/fpubh.2021.752509

Kostopoulou, O., Nurek, M., Cantarella, S., Okoli, G., Fiorentino, F., & Delaney, B. C. (2019). Referral Decision Making of General Practitioners: A Signal Detection Study. *Medical Decision Making*, *39*(1), 21–31. https://doi.org/10.1177/0272989X18813357

Kracht, L. W., & Heiss, W.-D. (2014). Metabolic Imaging. In M. Bernstein & M. S. Berger (Eds.), *Neuro-Oncology: The Essentials* (3rd ed.). Thieme Medical Publishers, Incorporated. http://ebookcentral.proquest.com/lib/gmul-ebooks/detail.action?docID=1790538

Loizidou, M., Sefcikova, V., Ekert, J. O., Bone, M., & Samandouras, G. (2022). Reforming support systems of newly diagnosed brain cancer patients: A systematic review. *Journal of Neuro-Oncology*, *156*(1), 61–71. https://doi.org/10.1007/s11060-021-03895-4

Lyratzopoulos, G., Wardle, J., & Rubin, G. (2014). Rethinking diagnostic delay in cancer: How difficult is the diagnosis? *BMJ*, *349*, g7400. https://doi.org/10.1136/bmj.g7400

McFaline-Figueroa, J. R., & Lee, E. Q. (2018). Brain Tumors. *The American Journal of Medicine*, *131*(8), 874–882. https://doi.org/10.1016/j.amjmed.2017.12.039

McNamara, S. (2011). Facing the challenges of primary malignant brain tumours. In T. N. Fawcett & A. McQueen (Eds.), *Perspectives on Cancer Care*. John Wiley & Sons, Incorporated. http://ebookcentral.proquest.com/lib/gmul-ebooks/detail.action?docID=624722

Mohammad, H. R., Boardman, J., Howell, L., Mills, R. J., & Emsley, H. C. A. (2016). Urgent referral for suspected CNS cancer: Which clinical features are associated with a positive predictive value of 3 % or more? *BMC Neurology*, *16*(1), 152. https://doi.org/10.1186/s12883-016-0677-1

Moher, D., Shamseer, L., Clarke, M., Ghersi, D., Liberati, A., Petticrew, M., Shekelle, P., Stewart, L. A., & PRISMA-P Group. (2015). Preferred reporting items for systematic review and meta-analysis protocols (PRISMA-P) 2015 statement. *Systematic Reviews*, *4*(1), 1. https://doi.org/10.1186/2046-4053-4-1

Nasreddine, Z. S., Phillips, N. A., Bédirian, V., Charbonneau, S., Whitehead, V., Collin, I., Cummings, J. L., & Chertkow, H. (2005). The Montreal Cognitive Assessment, MoCA: A

Brief Screening Tool For Mild Cognitive Impairment. *Journal of the American Geriatrics Society*, *53*(4), 695–699. https://doi.org/10.1111/j.1532-5415.2005.53221.x

NICE. (2015). *Recommendations organised by site of cancer | Suspected cancer: Recognition and referral | Guidance | NICE*. NICE. https://www.nice.org.uk/guidance/ng12/chapter/Recommendations-organised-by-site-of-cancer#brain-and-central-nervous-system-cancers

NICE. (2021). *NICE guideline: Brain tumours (primary) and brain metastases in over 16s*. 69.

NIHR. (2019). *Highlight Notice: Brain Tumours*. https://www.nihr.ac.uk/documents/highlight-notice-brain-tumours/11656

Office for National Statistics. (2018). *Cancer survival in England*. https://www.ons.gov.uk/peoplepopulationandcommunity/healthandsocialcare/conditionsanddiseases/bulletins/cancersurvivalinengland/stageatdiagnosisandchildhoodpatientsfollowedupto2018

Ozawa, M., Brennan, P. M., Zienius, K., Kurian, K. M., Hollingworth, W., Weller, D., Grant, R., Hamilton, W., & Ben-Shlomo, Y. (2019). The usefulness of symptoms alone or combined for general practitioners in considering the diagnosis of a brain tumour: A case-control study using the clinical practice research database (CPRD) (2000-2014). *BMJ Open*, *9*(8), e029686. https://doi.org/10.1136/bmjopen-2019-029686

Ozawa, M., Brennan, P. M., Zienius, K., Kurian, K. M., Hollingworth, W., Weller, D., Hamilton, W., Grant, R., & Ben-Shlomo, Y. (2018). Symptoms in primary care with time to diagnosis of brain tumours. *Family Practice*, *35*(5), 551–558. https://doi.org/10.1093/fampra/cmx139

Penfold, C., Joannides, A. J., Bell, J., & Walter, F. M. (2017). Diagnosing adult primary brain tumours: Can we do better? *British Journal of General Practice*, *67*(659), 278–279. https://doi.org/10.3399/bjgp17X691277

Perkins, A., & Liu, G. (2016). Primary Brain Tumors in Adults: Diagnosis and Treatment. *American Family Physician*, *93*(3), 211-217B.

Philips, A., Henshaw, D. L., Lamburn, G., & O'Carroll, M. J. (2018). Brain Tumours: Rise in Glioblastoma Multiforme Incidence in England 1995–2015 Suggests an Adverse Environmental or Lifestyle Factor. *Journal of Environmental and Public Health*, *2018*, e7910754. https://doi.org/10.1155/2018/7910754

Renovanz, M., Reitzug, L., Messing, L., Scheurich, A., Grüninger, S., Ringel, F., & Coburger, J. (2018). Patient reported feasibility and acceptance of Montreal Cognitive Assessment (MoCA) screening pre- and postoperatively in brain tumour patients. *Journal of Clinical Neuroscience*, *53*, 79–84. https://doi.org/10.1016/j.jocn.2018.04.034

Robinson, G. A., Biggs, V., & Walker, D. G. (2015). Cognitive Screening in Brain Tumors: Short but Sensitive Enough? *Frontiers in Oncology*, *5*. https://www.frontiersin.org/articles/10.3389/fonc.2015.00060

Sage, W., Fernández-Méndez, R., Crofton, A., Gifford, M. J., Bannykh, A., Chrysaphinis, C., Tingley, E., Bulbeck, H., Brahmbhatt, M., Pickard, J. D., Walter, F. M., Brodbelt, A., Price, S. J., & Joannides, A. J. (2019). Defining unmet clinical need across the pathway of brain tumor care: A patient and carer perspective. *Cancer Management and Research*, *11*, 2189–2202. https://doi.org/10.2147/CMAR.S175886

Salander, P., Bergenheim, A. T., Hamberg, K., & Henriksson, R. (1999). Pathways from symptoms to medical care: A descriptive study of symptom development and obstacles to early diagnosis in brain tumour patients. *Family Practice*, *16*(2), 143–148. https://doi.org/10.1093/fampra/16.2.143

Schünemann, H. J., Mustafa, R. A., Brozek, J., Steingart, K. R., Leeflang, M., Murad, M. H., Bossuyt, P., Glasziou, P., Jaeschke, R., Lange, S., Meerpohl, J., Langendam, M., Hultcrantz, M., Vist, G. E., Akl, E. A., Helfand, M., Santesso, N., Hooft, L., Scholten, R., … Guyatt, G. H. (2020). GRADE guidelines: 21 part 2. Test accuracy: inconsistency, imprecision, publication bias, and other domains for rating the certainty of evidence and presenting it in evidence profiles and summary of findings tables. *Journal of Clinical Epidemiology*, *122*, 142–152. https://doi.org/10.1016/j.jclinepi.2019.12.021

Scott, S. E., Penfold, C., Saji, S., Curtis, S., Watts, C., Hamilton, W., Joannides, A. J., & Walter, F. M. (2019). 'It was nothing that you would think was anything': Qualitative analysis of appraisal and help seeking preceding brain cancer diagnosis. *PLOS ONE*, *14*(3), e0213599. https://doi.org/10.1371/journal.pone.0213599

Senan, E. M., Jadhav, M. E., Rassem, T. H., Aljaloud, A. S., Mohammed, B. A., & Al-Mekhlafi, Z. G. (2022). Early Diagnosis of Brain Tumour MRI Images Using Hybrid Techniques between Deep and Machine Learning. *Computational and Mathematical Methods in Medicine*, *2022*, e8330833. https://doi.org/10.1155/2022/8330833

Smith, R. (2002). In search of "non-disease". *BMJ : British Medical Journal*, *324*(7342), 883–885.

Tessa Jowell Brain Cancer Mission. (2019, August 12). *Tessa Jowell Brain Cancer Mission (TJBCM) | Calling for action to improve brain tumour treatment, research and survival.* Tessa Jowell Brain Cancer Mission (TJBCM) | Calling for Action to Improve Brain Tumour Treatment, Research and Survival. https://www.tessajowellbraincancermission.org/

Villalobos, D., Torres-Simón, L., Pacios, J., Paúl, N., & del Río, D. (2022). A Systematic Review of Normative Data for Verbal Fluency Test in Different Languages. *Neuropsychology Review*. https://doi.org/10.1007/s11065-022-09549-0

Vuralli, D., Ayata, C., & Bolay, H. (2018). Cognitive dysfunction and migraine. *The Journal of Headache and Pain*, *19*(1), 109. https://doi.org/10.1186/s10194-018-0933-4

Walsh, K. M., Claus, E. B., & Wrensch, M. R. (2014). Epidemiology. In M. Bernstein & M. S. Berger (Eds.), *Neuro-Oncology: The Essentials* (3rd ed.). Thieme Medical Publishers, Incorporated. http://ebookcentral.proquest.com/lib/gmul-ebooks/detail.action?docID=1790538

Walter, F. M., Penfold, C., Joannides, A., Saji, S., Johnson, M., Watts, C., Brodbelt, A., Jenkinson, M. D., Price, S. J., Hamilton, W., & Scott, S. E. (2019). Missed opportunities for diagnosing brain tumours in primary care: A qualitative study of patient experiences. *British Journal of General Practice*, *69*(681), e224–e235. https://doi.org/10.3399/bjgp19X701861

Wilkinson, I. M. S., Lennox, Graham. (2005). *Essential neurology*. Wiley-Blackwell.

Zhou, Y., Mendonca, S. C., Abel, G. A., Hamilton, W., Walter, F. M., Johnson, S., Shelton, J., Elliss-Brookes, L., McPhail, S., & Lyratzopoulos, G. (2018). Variation in 'fast-track' referrals for suspected cancer by patient characteristic and cancer diagnosis: Evidence from 670 000 patients with cancers of 35 different sites. *British Journal of Cancer*, *118*(1), Article 1. https://doi.org/10.1038/bjc.2017.381

Zienius, K., Chak-Lam, I., Park, J., Ozawa, M., Hamilton, W., Weller, D., Summers, D., Porteous, L., Mohiuddin, S., Keeney, E., Hollingworth, W., Ben-Shlomo, Y., Grant, R., & Brennan, P. M. (2019). Direct access CT for suspicion of brain tumour: An analysis of referral pathways in a population-based patient group. *BMC Family Practice*, *20*(1), 118. https://doi.org/10.1186/s12875-019-1003-y

Appendices

Appendix A

Systematic Review Search Strategy

**Cognitive function tests that discriminate between patients with and without brain tumour**		
Numbers 1 and 2 are the keywords and MeSH terms in the title or abstract for the **Population** and are combined using **OR**	1. Brain tumour* or brain cancer*<br><br>2. "Brain Neoplasms"[Mesh]	Population (P)
Numbers 3 and 4 are the keywords and MeSH terms in the title or abstract for the **Intervention** and are combined using **OR**	3. Cognit* function* test* OR cognit* function* assessment* OR cognitive function* exam* OR executive function* test* OR executive function* assessment* OR executive function* exam* OR neuropsycholog* assessment* OR neuro-psycholog* assessment* OR neuropsycholog* exam* OR neuro-psycholog* exam* OR cognit* test* OR cognit* assessment* OR cognit* exam* OR cognit* abilit* test* OR clock-drawing test OR Montreal cognitive test OR mini-mental state exam OR abbreviated mental test OR memory impairment screen OR mental status questionnaire OR short portable mental status questionnaire OR neuropsychiatric inventory questionnaire OR mini examen cognoscitivo OR Eurotest OR Fototest OR memory alteration test OR verbal fluency OR memory OR mental capacity	Intervention (I)

	4. "Neuropsychological Tests"[Mesh]	
**P** and **I** keywords and MeSH terms combined using **AND**		P and I combined

Pilot Search

MEDLINE (via PubMed)

((Brain tumour*[Title/Abstract]) OR (brain cancer*[Title/Abstract]) OR (brain neoplasms[MeSH Terms])) AND ((Cognit* function* test*[Title/Abstract]) OR (cognit* function* assessment*[Title/Abstract]) OR (cognitive function* exam*[Title/Abstract]) OR (executive function* test*[Title/Abstract]) OR (executive function* assessment*[Title/Abstract]) OR (executive function* exam*[Title/Abstract]) OR (neuropsycholog* assessment*[Title/Abstract]) OR (neuro-psycholog* assessment*[Title/Abstract]) OR (neuropsycholog* exam*[Title/Abstract]) OR (neuro-psycholog* exam*[Title/Abstract]) OR (cognit* test*[Title/Abstract]) OR (cognit* assessment*[Title/Abstract]) OR (cognit* exam*[Title/Abstract]) OR (cognit* abilit* test*[Title/Abstract]) OR (clock-drawing test[Title/Abstract]) OR (Montreal cognitive test[Title/Abstract]) OR (mini-mental state exam[Title/Abstract]) OR (abbreviated mental test[Title/Abstract]) OR (memory impairment screen[Title/Abstract]) OR (mental status questionnaire[Title/Abstract]) OR (short portable mental status questionnaire[Title/Abstract]) OR (neuropsychiatric inventory questionnaire[Title/Abstract]) OR (mini examen cognoscitivo[Title/Abstract]) OR (Eurotest[Title/Abstract]) OR (Fototest[Title/Abstract]) OR (memory alteration test[Title/Abstract]) OR (verbal fluency[Title/Abstract]) OR (memory[Title/Abstract]) OR (mental capacity[Title/Abstract]) OR (neuropsychological test[MeSH Terms]))

3324 results

CENTRAL

(Mesh brain neoplasms) OR brain tumour* or brain cancer* in Title Abstract Keyword AND (Mesh neuropsychological tests) OR cognit* function* test* OR cognit* function* assessment* OR cognitive function* exam* OR executive function* test* OR executive function* assessment* OR executive function* exam* OR neuropsycholog* assessment* OR neuro-psycholog* assessment* OR neuropsycholog* exam* OR neuro-psycholog* exam* OR cognit* test* OR cognit* assessment* OR cognit* exam* OR cognit* abilit* test* OR clock-

drawing test OR Montreal cognitive test OR mini-mental state exam OR abbreviated mental test OR memory impairment screen OR mental status questionnaire OR short portable mental status questionnaire OR neuropsychiatric inventory questionnaire OR mini examen cognoscitivo OR Eurotest OR Fototest OR memory alteration test OR verbal fluency OR memory OR mental capacity in Title Abstract Keyword - (Word variations have been searched)
1322 total results:

EMBASE (with all synonyms included)

('brain tumor'/exp OR 'brain neoplasm' OR 'brain neoplasms' OR 'brain supratentorial tumor' OR 'brain supratentorial tumour' OR 'brain tumor' OR 'brain tumor diagnosis' OR 'brain tumour' OR 'brain tumour diagnosis' OR 'cerebral tumor' OR 'cerebral tumour' OR 'cerebroma' OR 'cerebrum tumor' OR 'cerebrum tumour' OR 'encephalophyma' OR 'intracerebral tumor' OR 'intracerebral tumour' OR 'intracranial neoplasm' OR 'midline tumor' OR 'midline tumour' OR 'multiple brain tumor' OR 'multiple brain tumour' OR 'subtentorial tumor' OR 'subtentorial tumour' OR 'supratentorial brain tumor' OR 'supratentorial brain tumour' OR 'supratentorial neoplasms' OR 'supratentorial tumor' OR 'supratentorial tumour' OR 'tumor cerebri' OR 'tumor, brain' OR 'tumour cerebri' OR 'tumour, brain' OR 'brain cancer'/exp OR 'brain cancer' OR 'brain carcinoma' OR 'brain malignant tumor' OR 'brain malignant tumour' OR 'carcinoma, brain' OR 'carcinoma, cerebral' OR 'cerebral carcinoma' OR 'cerebral neoplasm') AND ('cognitive function test'/exp OR 'cognition test' OR 'cognitive abilities test' OR 'cognitive ability test' OR 'cognitive function test' OR 'cognitive functioning test' OR 'cognition assessment'/exp OR 'cognition assessment' OR 'cognitive function assessment' OR 'memory and learning tests' OR 'executive function test'/exp OR 'executive function task' OR 'executive function test' OR 'problem solving task' OR 'problem solving test' OR 'neuropsychological test'/exp OR 'neuro-psychological assessment' OR 'neuro-psychological examination' OR 'neuro-psychological test' OR 'neuropsychologic test' OR 'neuropsychological assessment' OR 'neuropsychological examination' OR 'neuropsychological test' OR 'neuropsychological tests' OR 'neuropsychology test' OR 'test, neuropsychological' OR 'cognitive assessment'/exp OR 'clock drawing test'/exp OR 'montreal cognitive assessment'/exp OR 'montreal cognitive assessment' OR 'mini mental state examination'/exp OR 'mmse' OR 'mini mental state examination' OR 'mini-mental state examination' OR 'abbreviated mental test'/exp OR 'memory impairment screen'/exp OR 'short portable mental status questionnaire'/exp OR 'spmsq' OR 'short portable mental status questionnaire' OR 'short portable mental status examination' OR 'short portable mental status score' OR 'neuropsychiatric inventory questionnaire'/exp OR 'memory alteration test'/exp OR

'verbal fluency'/exp OR 'memory'/exp OR 'item recall' OR 'memory' OR 'memory function' OR 'nonspatial memory' OR 'remembering' OR 'reminiscence' OR 'mental capacity'/exp OR 'ability, mental' OR 'attainment' OR 'capacity, mental' OR 'fitness, mental' OR 'mental ability' OR 'mental capacity' OR 'mental competency' OR 'mental fitness')

6043 results
